# Harm reduction in the USA: the research perspective and an archive to David Purchase

**DOI:** 10.1186/s12954-017-0178-6

**Published:** 2017-07-26

**Authors:** Don C. Des Jarlais

**Affiliations:** The Baron Edmond de Rothschild Chemical Dependency Institute, Icahn School of Medicine at Mount Sinai, 39 Broadway Suite 530, New York, NY 10006 USA

**Keywords:** HIV, Persons who inject drugs, Syringe exchange, Harm reduction

## Abstract

The history of harm reduction in the USA has led to the development of some of the most important methods for treating persons for drug use disorders, such as methadone and buprenorphine for opiate use disorder. However, there has been fierce political resistance to implementation and scale-up of harm reduction in the USA. This resistance is rooted in historical demonization of particular psychoactive drugs that were associated with stigmatized racial/ethnic groups.

With the discovery of acquired immunodeficiency syndrome (AIDS) in 1981, harm reduction became important not only for treating substance use disorders, but for reducing transmission of blood-borne infection. However, within the context of the crack cocaine epidemic in the 1980s, it was very difficult to implement any programs that appeared to “condone” drug use.

It was not until the late 1980s that syringe exchange programs began at the state and local level in the USA. With funding primarily from state and local governments and the support of the North American Syringe Exchange Network (NASEN), there are now approximately 200 programs for syringe exchange in the USA. Research has shown that these programs have been extremely effective in reducing human immunodeficiency virus (HIV) transmission among persons who inject drugs (PWID). The programs in the USA also offer many additional services for drug users, including condom distribution, referrals to substance abuse treatment, HIV, hepatitis C virus (HCV), hepatitis B virus (HBV) counseling and testing, overdose education and naloxone distribution to reverse for overdose.

Currently, the USA is experiencing an opioid/heroin epidemic, with significant increases in overdose deaths among drug users. Much of this epidemic is occurring in suburban and rural of the country without harm reduction services. The current challenges for harm reduction and harm reduction research involve expansion of services to suburban and rural areas and implementation science on how to effectively and efficiently address HCV transmission and overdose. Most importantly, continued research efforts are needed to reduce the stigma of psychoactive drug use. While political opposition continues, harm reduction activists and researchers have developed a highly effective partnership based on a common core values.

**Electronic supplementary material:**

The online version of this article (doi:10.1186/s12954-017-0178-6) contains supplementary material, which is available to authorized users.

## Introduction: harm reduction and harm reduction research

Harm reduction has a very complex history in the USA. The USA has led the world in developing some aspects of harm reduction, e.g., methadone- and buprenorphine-assisted treatment for opiate use disorders, but the US federal government was for a long time a fierce opponent of harm reduction both domestically and internationally. The history of harm reduction in the USA is best understood as a conflict between multiple conflicting social/historical forces.

The two most important forces promoting harm reduction in the USA have been activism and scientific research. The role of activism has been very well described by Moore and Clear [1].

There has been great cooperation between harm reduction activists and HIV/AIDS researchers over the last three decades. The activists were typically in the forefront of the struggle to implement harm reduction. The researchers then provided the data needed to justify large-scale public expenditures on harm reduction programs (primarily by state and local governments). Without these public expenditures, the harm reduction programs would not have achieved the scope they needed to be successful to stop the HIV epidemic among people who inject drugs (PWID). This paper will focus on the contributions of scientific research to harm reduction in the USA, with an acknowledgement that without the contributions of the activists, harm reduction programs would not have been created, and the research supporting harm reduction could not have occurred.

## Historical background

Before discussing harm reduction research in the USA, it will be helpful to provide some historical context. First, the USA has a long tradition of moralistic condemnation of intoxication with psychoactive drugs (including licit drugs like alcohol). The Puritans are often blamed for this tradition, even though they consumed alcohol (often in considerable quantities) [2, 3]. They did, however, condemn inebriation and also set precedents for extensively incorporating religious codes into civil laws.

A second important historical component of the harm reduction struggle in the USA has been the demonization of the psychoactive drugs associated with stigmatized racial/ethnic minority groups. This includes the use of opium by Chinese immigrants [4], the use of cocaine by African-Americans [5], and the use of marijuana by Mexican-Americans [6]. The combination of moralistic intolerance of intoxication and stigmatization of minority groups often led to the demonization of many psychoactive drugs. This demonization of specific drugs did not prevent the use of the drugs, but it did create a context in which the drugs were feared, there was fear of and anger towards the drug users, and abstinence was seen as the only acceptable policy towards drug use. Public discourse about illicit drug users was characterized by stereotypes of both the drugs and the users. Criminal law was viewed as the most appropriate means for controlling drug use.

The third important background factor for the harm reduction struggle was the federal system of government in the USA. The individual states have great responsibilities, including for public health. Thus, individual states could implement harm reduction programs in opposition of the attitudes of the federal government. The federal government, however, had much greater financial resources for both implementing HIV prevention for PWID and for funding research. Thus, the initial opposition to harm reduction by the US federal government delayed widespread implementation of harm reduction programs for many years.

The final component of the historical context was the US tradition of biomedical and health research. After World War II, the USA became the world leader in biomedical and health research, with the US government as the dominant funder of this research. There was a general respect for science in American society and an expectation that scientific research could and should be applied to solve societal problems, particularly health-related problems.

The science of psychoactive drug use and substance use disorders at the time AIDS was first observed among PWID was still in a very early stage [7]. Most drug researchers generally viewed substance use disorders as a disease—in contrast to the general public, which tended to view substance use disorders as a moral failing. The great majority of scientists studying substance use disorders in the 1950s through the 1980s, however, believed that the only solution to the problems of drug use was total abstinence from drug use.

The most notable exception to this general view of substance use disorders was the development of methadone maintenance by Dole and Nyswader [8]. Methadone maintenance was harm reduction in that it showed the possibility of reducing both individual and societal problems associated with drug use without requiring that users cease all psychoactive drug use. The great insights of methadone maintenance were that many of the problems created by drug use were related to the particular characteristics of the individual drugs (duration of effect, route of administration), and that some psychoactive drugs could be used as very effective medication for treating drug use disorders.

## The introduction, unseen spread, and by the discovery of HIV among PWID in the USA

AIDS was first observed among PWID in the USA in 1981, several months after being first observed among men who have sex with men (MSM) [9]. The initial observations were made on relatively small numbers of PWID with AIDS and were almost exclusively confined to the northeastern USA. It was not until the development of the HIV antibody test in 1984–1985 and large-scale antibody testing for HIV that the full scope of the problem was revealed. HIV had been spreading among PWID in New York City and in many other USA and European cities during this time. HIV prevalence had reached 50% in New York City, and the virus was present among PWID in many other USA and Western European cities [10, 11].

There were several aspects of the timing of the first observation that made it extremely difficult to combat this new epidemic. First, as noted above, HIV had already spread in the USA and in many other countries before AIDS was first observed among PWID, so that it was already too late to prevent the initial spread of the virus. Second, important changes in the politics and in the epidemiology of drug use had recently occurred. In the late 1970s, there was a movement to decriminalize possession for personal use of marijuana in some states, and even consideration of decriminalization of cocaine possession for personal use [12]. This changed with the election of Ronald Reagan, in 1980, who adopted a “just say no” attitude towards drug use.

Another major factor was the emergence of the large-scale crack cocaine epidemic [13]. Not only did the use of crack cocaine increase greatly among inner city African-American neighborhoods but there was a great deal of violence associated with the distribution of the drug. The crack cocaine epidemic and the fear of drug use-related violence tapped into the long-standing stigmatization of African-Americans and the demonization of psychoactive drugs. The crack cocaine epidemic made it difficult for political leaders to support any programs such as syringe exchange that appeared to “encourage” or “condone” illicit drug use. Much of the African-American community, which experienced both the negative effects of the crack epidemic and increased stigmatization due to the crack epidemic, was adamantly opposed to syringe exchange programs.

## The science of initial efforts at syringe exchange harm reduction and the pseudo-science of opposition syringe exchange in the USA

When HIV antibody testing was first implemented in Amsterdam, the prevalence was over 30% [14]. The city had already implemented a small syringe exchange program (also referred to as needle and syringe exchange programs, syringe service programs, syringe access programs, syringe distribution programs, needle/syringe exchange programs) the year before in an effort to reduce the transmission of hepatitis B virus (HBV) among PWID. This exchange program was started after a large pharmacy in the central city stopped selling needles and syringes to drug users. With the discovery of the very substantial HIV/AIDS problem among PWID in the city, the Amsterdam health department rapidly expanded the exchange program, and other Dutch cities implemented programs.

HIV antibody testing among PWID in the UK found a high prevalence epidemic in Edinburgh, Scotland, moderate prevalence in London, and generally low prevalence in other areas. The health department sent a delegation to New York City to learn more about HIV/AIDS among PWID. The health department then set up pilot syringe exchange programs in multiple cities, did a rapid evaluation of the pilot programs, and then expanded to a public health level. The pilot program evaluation in the UK did lead to some important findings, specifically that the programs needed to be “user friendly” in order to be successful.

### Early attempts at syringe exchange research, early syringe exchange research, and early resistance to syringe exchange research

The idea of setting up pilot programs, evaluating them, and then expanding them based on the evaluation finding was also attempted in the USA, but with considerable difficulties. In 1985, the New York City Department of Health proposed a pilot program, but the memorandum from the Commissioner of Health to the Mayor was leaked to the police, who effectively vetoed the proposal. A pilot project with an evaluation component was then proposed and adopted in 1988 despite very strong opposition [15]. The project was described by one opponent as “genocide” for the African-American community. The pilot project did produce positive results in getting PWID into long-term substance use treatment [16] but was not large enough to have an effect on unsafe injection and HIV transmission. The pilot project was discontinued in 1989.

Other attempts in the late 1980s at conducting syringe exchange research in Tacoma, Washington, and New Haven, Connecticut, were more successful. The Tacoma research documented reduced risk behavior [17] and lower HBV incidence [18] among syringe exchange participants compared to non-participants [19]. The New Haven program was evaluated using a mathematical model of HIV transmission based on the reduction over time of the presence HIV antibody in syringes returned to the exchange. The decline in HIV antibody presence in the syringes returned to the exchange indicated a reduction in syringe sharing and thus a likely reduction in HIV transmission [20]. It is notable that these early research efforts were funded by private foundations—the American Foundation for AIDS Research (amFAR) for the Tacoma studies and the Robert Wood Johnson Foundation (RWJF) for the New Haven studies. AmFAR later funded a very large study in New York City [21]. (The Comer Foundation was also important for their very early support of syringe exchange in the USA.)

In 1988, opponents of syringe exchange added a provision to the funding bill for the Department of Health and Human Services (HHS) that prohibited the use of federal funds for supporting syringe exchange programs until the Secretary of HHS found that syringe exchange programs were “safe and effective.” This wording was parallel to the federal Food and Drug Administration (FDA) requirement that new drugs be shown to be safe and effective before they are approved for sale in the USA.

There were, however, critical differences. At the same time, the federal government was refusing to fund research on syringe exchange. As the predominant funder of research on preventing HIV infection, the lack of federal funding for syringe exchange research created a catch-22 that delayed showing safety and effectiveness of syringe exchange for many years. Federal funds could not be used to support syringe exchange until research showed that syringe exchange was “safe and effective,” but without federal funding of syringe exchange, there were very few programs that could be researched and very little money for conducting research—the federal government was at the time also refusing to fund research on syringe exchange.

Fortunately, as noted above, several private foundations did fund syringe exchange research, in particular amFAR and RWJF.

### The epidemic continues, research accelerates and accumulates, but the federal ban continues

The debates on syringe exchange and conducting research on syringe exchange were occurring within the context of greatly increasing numbers of HIV/AIDS cases among PWID during the early to mid-1990s. This led increasing numbers of state and local governments, activist groups, and private foundations to implement programs. The North American Syringe Exchange Network (NASEN) grew rapidly from approximately 50 programs in 1995 to over 100 programs by 1997 [22]. The increase in the numbers of programs provided many more opportunities for conducting research. NASEN also collaborated with the Chemical Dependency Institute of Beth Israel Medical Center to conduct annual surveys of syringe exchange programs in the USA. These surveys provided the only national data on programs and were often published in the Centers for Disease Control Morbidity and Mortality Weekly report (CDC MMWR) [23, 24]. We do not yet have a formal written history of NASEN, but the online Appendix for this paper contains a number of press reports of NASEN activities (Additional file 1).

The National Institute on Drug Abuse (NIDA) also began funding research on syringe exchange programs. This then lead to a large increase in the number of scientific papers published on syringe exchange in the USA. There were only 32 articles on syringe exchanged in the USA published prior to 1995, there were 63 by 1996, 150 by 2000, and approximately 560 by early 2017 [25].

The growth of research on syringe exchange in the USA, and in other countries, led to a number of important policy statements and scientific literature reviews on the topic. These included the “Twin Epidemics” report by the US National Commission on AIDS [26], a Centers for Disease Control review [23, 24] and two National Academies of Science/Institute of Medicine reports [27, 28] All of these supported the use of syringe exchange as a method for reducing HIV transmission among PWID.

By 1998 the scientific evidence in support of syringe exchange was sufficiently compelling that the Secretary of Health and Human Services did make the finding that syringe exchange was “safe and effective.” The opposition to the syringe exchange was still sufficiently strong in the US Congress, however, that the Clinton administration did not attempt to approve federal funding for syringe exchange programs, knowing that permitting use of federal funds would be rejected by Congress. This clearly demonstrated that it was political opposition to the syringe exchange and not the lack of scientific evidence on safety and effectiveness that was keeping the ban in place. (For further discussion of the relationships between the data on the effectiveness of syringe exchange and the lack of policy change, see Allen et al. [29] and Blankenship et al. [30, 31].)

## The evolution of syringe exchange programs and syringe exchange research

While the original purpose of syringe exchange programs was to reduce transmission of blood-borne infections among PWID, programs in the USA rapidly evolved into multi-service organizations. In addition to basic syringe exchange, the programs have provided a wide range of additional health and social services to people who use drugs (and also often to community members who do not use drugs). These services include condoms, referrals to substance abuse treatment, HIV, hepatitis C virus (HCV), HBV counseling and testing, and naloxone for overdose [32]. Of particular importance is education about overdose and the distribution of naloxone to drug users, their friends and families for reversing overdoses.

A second major direction may be termed operations or implementation research—how to effectively and efficiently provide services within limited resources. Innovative operational components have included syringe distribution without requiring one-for-one exchanges, secondary exchange/peer-delivered exchange in which program participants exchange for other drug users who do not personally attend the program, and individually scheduled exchange in which program staff meet participants at agreed upon times and locations to conduct exchange outside of the regular program hours and locations. As noted below, such operations/implementation research has become critical for addressing the current opioid/heroin epidemic in the USA.

## The current situation and current harm reduction research in the USA

Since 2002, the USA has been experiencing an opioid/heroin epidemic [33, 34]. A large increase in the prescription of opioid analgesics to treat pain was followed by large increase in the numbers of persons who became addicted to opioids and who then transitioned from oral opioid use to injection of opioids and heroin [35]. This new opioid/heroin epidemic can most readily be seen in the increase in the number of drug overdose deaths in the USA, from 16,849 in 2002 to 52,404 in 2015 (over a threefold increase) [36]. Much of the increase in opioid/heroin use resulted from over-prescribing of opioid analgesics, and has occurred in suburban and rural areas of the country where medication-assisted treatment and syringe exchange services were lacking.

The opioid/heroin epidemic also led to an outbreak of HIV in Scott County, Indiana. With a population of only 23,744, Scott County experienced 181 new HIV infections in 2015 [37]. At the time of the outbreak, syringe exchange was illegal in the state of Indiana. In response to this public health catastrophe, syringe exchange was eventually legalized in Indiana and other neighboring states [38] and the ban on using federal funds for syringe exchange programs was effectively lifted [39]. The symbolic content of the ban was retained, however, in that federal funds could not be used for the purchase of needles and syringes, but could be used for staff, rent, services in addition to exchange and the other expenses needed to successfully operate a program. The cost of the needles and syringes is quite modest in comparison to the other costs, so this symbolic restriction is often not of great practical importance for most programs. The lifting of the ban on using federal funds did not include any new funds, it merely permitted state governments to re-allocate federal funds to support some of the expenses in operating syringe exchange programs. Many programs in the USA remain underfunded [23], and funding for the programs will need to be substantially increased to meet the challenges of the new opioid epidemic.

Research on harm reduction programs over the last 30 years in the USA has unequivocally demonstrated that these programs can minimize HIV transmission among PWID [40]. The current challenges for harm reduction and research on harm reduction involve reducing overdoses, reducing HCV transmission among PWID, including providing treatment for hepatitis infection to persons already infected. There are major issues in implementation research for providing harm reduction services to persons who use drugs in suburban and rural areas. These areas typically do not have current services, and there are transportation and economic difficulties for providing the needed services.

The greatest research issue for improving harm reduction in USA is identifying ways of reducing the intense stigmatization of persons who use many different psychoactive drugs. Psychoactive drug use can certainly generate many severe individual and societal harms but the severe stigmatization of persons using the drugs contributes rather than ameliorates the individual and societal harms.

## The common values in harm reduction services and research on harm reduction

The two basic components of harm reduction are pragmatism—providing policies and services that are effective—and respect for the human rights of persons who use drugs. The two basic components of research on harm reduction are measuring the harm that can be reduced through improved policies and new programs and conducting ethical research with persons who use drug. Ethical research with human subjects involves (1) benevolence, the research should benefit the persons who participated as subjects, (2) autonomy, the participants should have the right to determine what activities they will and will not participate in, and (3) justice, the research should benefit not just the individual participants but also the community of the persons who participated [41]. Both harm reduction and research on harm reduction in the USA were restricted by political opposition, but still formed a highly effective partnership based on their common values.

## Conclusions

1. The history of harm reduction in the USA reflects multiple competing components of US society, including moralistic condemnation of intoxication and of dependence on psychoactive drugs, stigmatization of racial/ethnic minority groups and demonization of the psychoactive drugs used by particular minority groups, and a tradition of using science to address health problems.
2. The discovery of AIDS among persons who inject drugs in the USA, came after the HIV virus had already spread rapidly in some parts of the country (so that primary prevention was not possible in those areas) and in the context of a crack cocaine epidemic, which made it very difficult to establish a public health approach to HIV/AIDS among persons who injected drugs.
3. Activists initiated syringe exchange programs in several parts of the country, and researchers collected sufficient data to convince state and local governments to provide the funding needed for large-scale implementation of syringe exchange program.
4. Syringe exchange has been remarkably effective in reducing HIV transmission, and most programs now address many addition health and social needs of their participants
5. Harm reduction programming and harm reduction research share two critical values: identifying what is pragmatically effective and respecting the human rights of persons who use drugs.

## Additional file

Additional file 1: Dave Purchase Press Stories Collection Appendix. (DOC 78422 kb)

## Abbreviations

AIDS: Acquired immunodeficiency syndrome
amFAR: American Foundation for AIDS Research
CDC MMWR: Centers for Disease Control Morbidity and Mortality Weekly report
FDA: Food and Drug Administration
HBV: Hepatitis B virus
HCV: Hepatitis C virus
HHS: Health and Human Services
HIV: Human immunodeficiency virus
MSM: Men who have sex with men
NASEN: North American Syringe Exchange Network
NIDA: National Institute on Drug Abuse
PWID: Persons who inject drugs
RWJF: Robert Wood Johnson Foundation
