# Supplementary material for: Harm reduction in the USA: the research perspective and an archive to David Purchase
Source: Harm Reduct J. 2017 Jul 26;14:51. doi: 10.1186/s12954-017-0178-6 (PMC5530540; doi:10.1186/s12954-017-0178-6)

**Introduction to the Dave Purchase Press Stories Collection Appendix**

Don C. Des Jarlais

I first met Dave Purchase at the National HIV Conference in 1989. I had just finished a plenary address on HIV among persons who inject drugs when Dave and Holly Hagan came up to me and asked if I would help in conducting research on the Tacoma syringe exchange program. Dave had started the program and Holly was the local Pierce County epidemiologist. Based on his personal experience working with persons who inject drugs and his experience in founding and operating the program, Dave was certain of the effectiveness of syringe, but he wanted rigorous research to formally demonstrate the effectiveness of the programs and to provide information on how to improve the programs.

That initial meeting led my becoming a founding member of the board of directors for the North American Syringe Exchange Network and to decades of highly productive collaborative research between Dave and myself. The research included the initial studies of the Tacoma program with Holly Hagan, and the later national surveys of syringe exchange programs in the US, which were conducted in conjunction with NASEN. This survey has provided the only data on the operations of exchanges in the US, and the results have been frequently published in the Centers for Disease Control Morbidity and Mortality Weekly Report (MMWR). This survey has been renamed the “Dave Purchase Memorial Syringe Exchange Survey.”

In writing this article for Harm Reduction Journal, I had originally hoped to include a formal history of NASEN. Time and other constraints did not permit this, but NASEN had collected a number of press stories about Dave and the organization. I hope that these stories provide additional insight into the life of a person who has done as much as anyone to promote the health and well-being of persons who use drugs and the organization that he founded.

**Additional file I**

**List of press releases/news articles referencing NASEN (North American Syringe Exchange Network) or Syringe Exchange in United States (with special focus on Dave Purchase)**
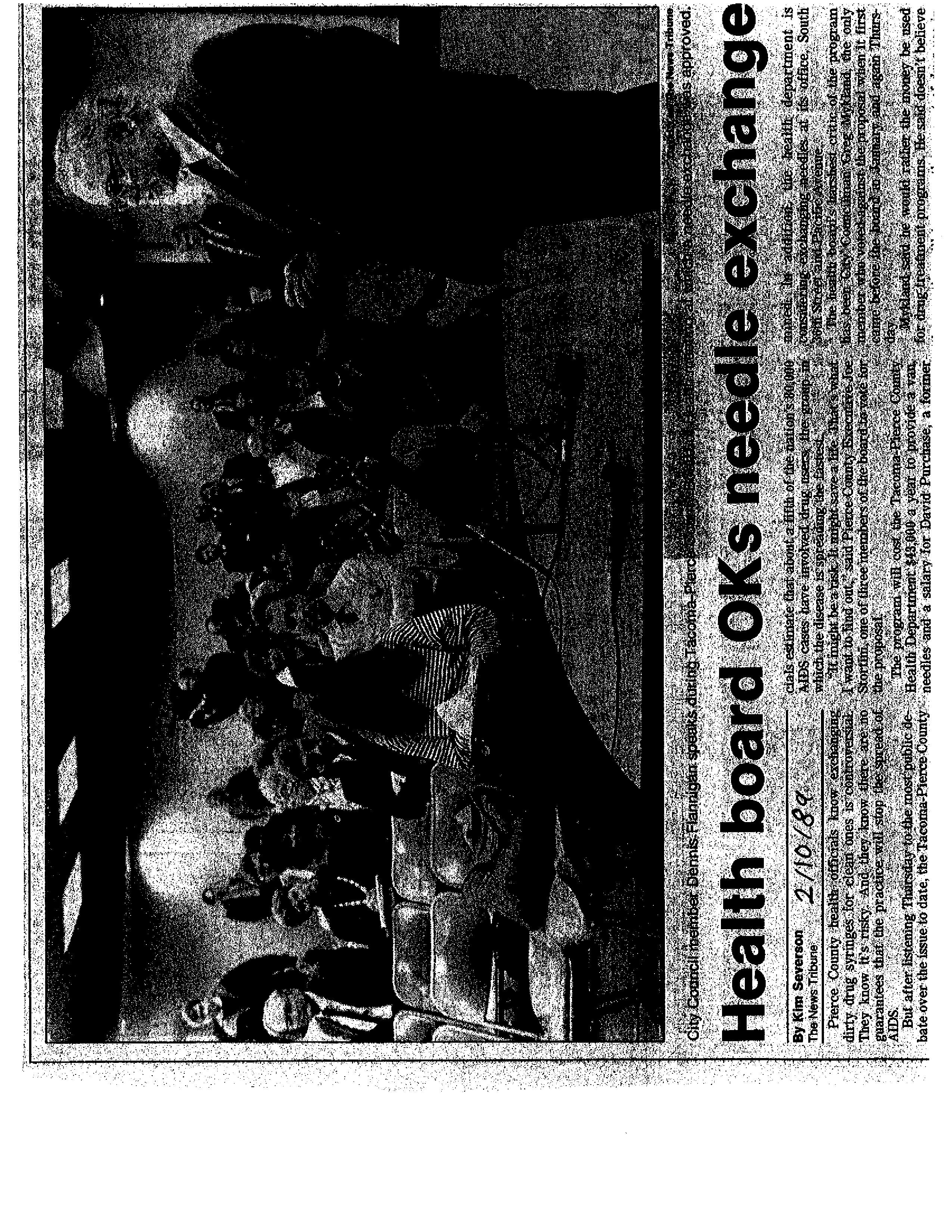

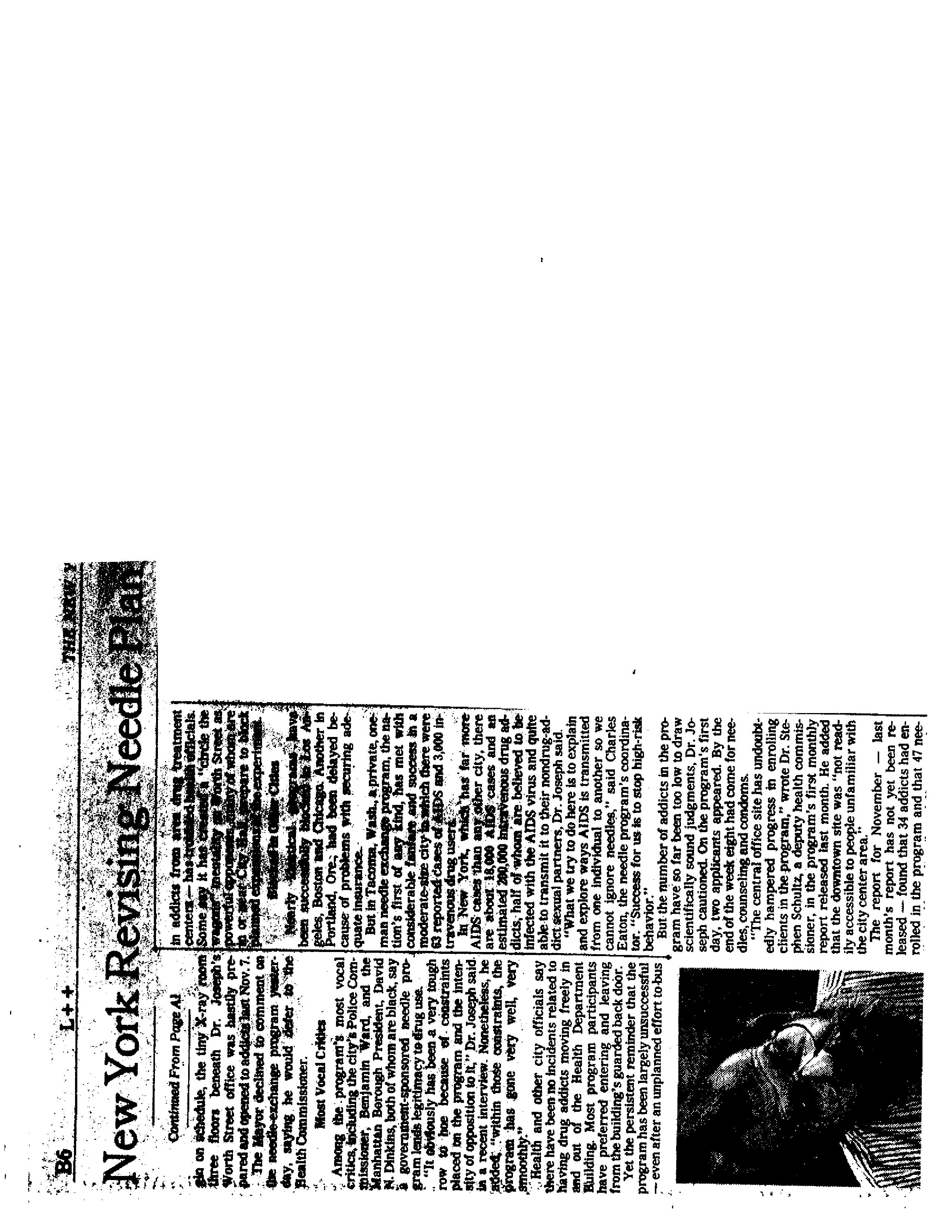

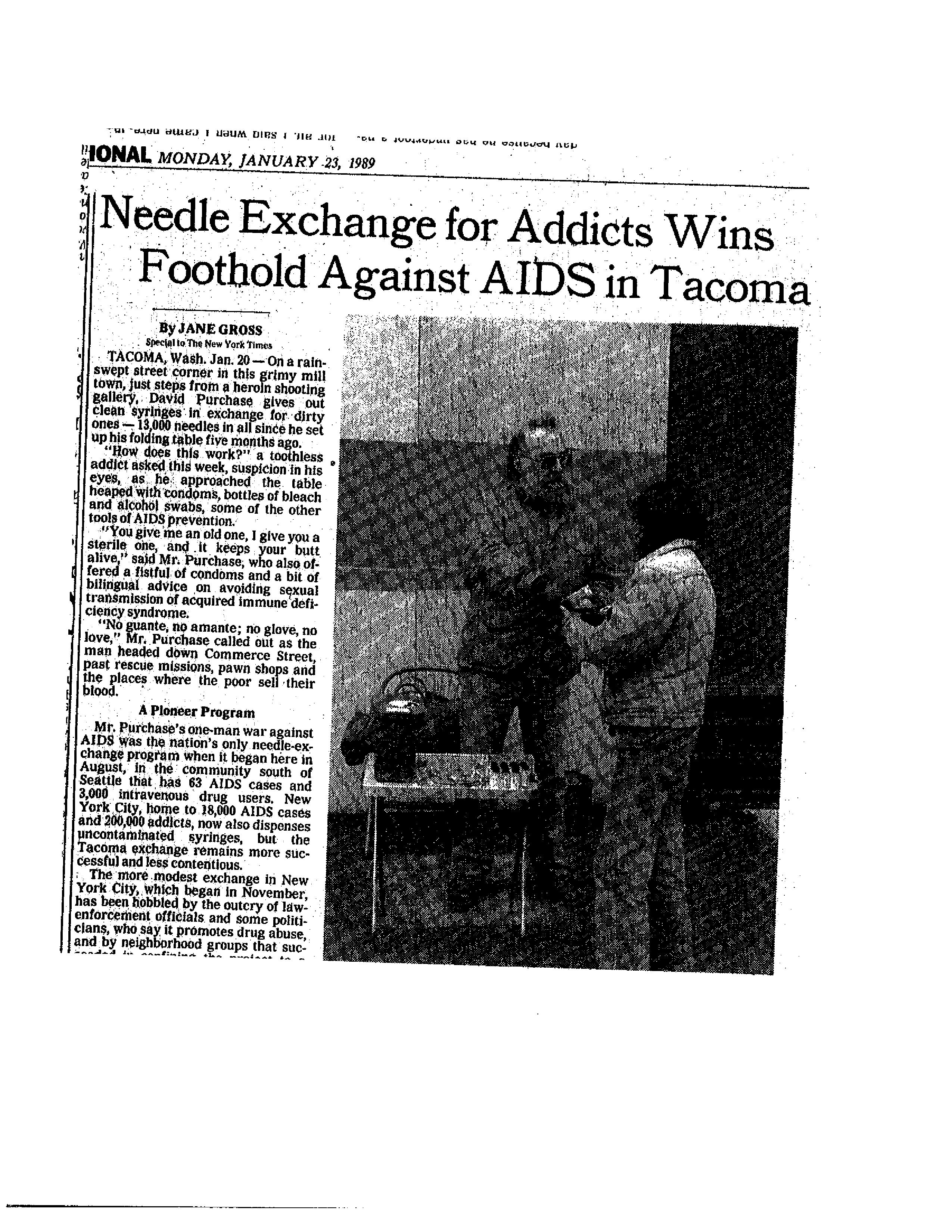

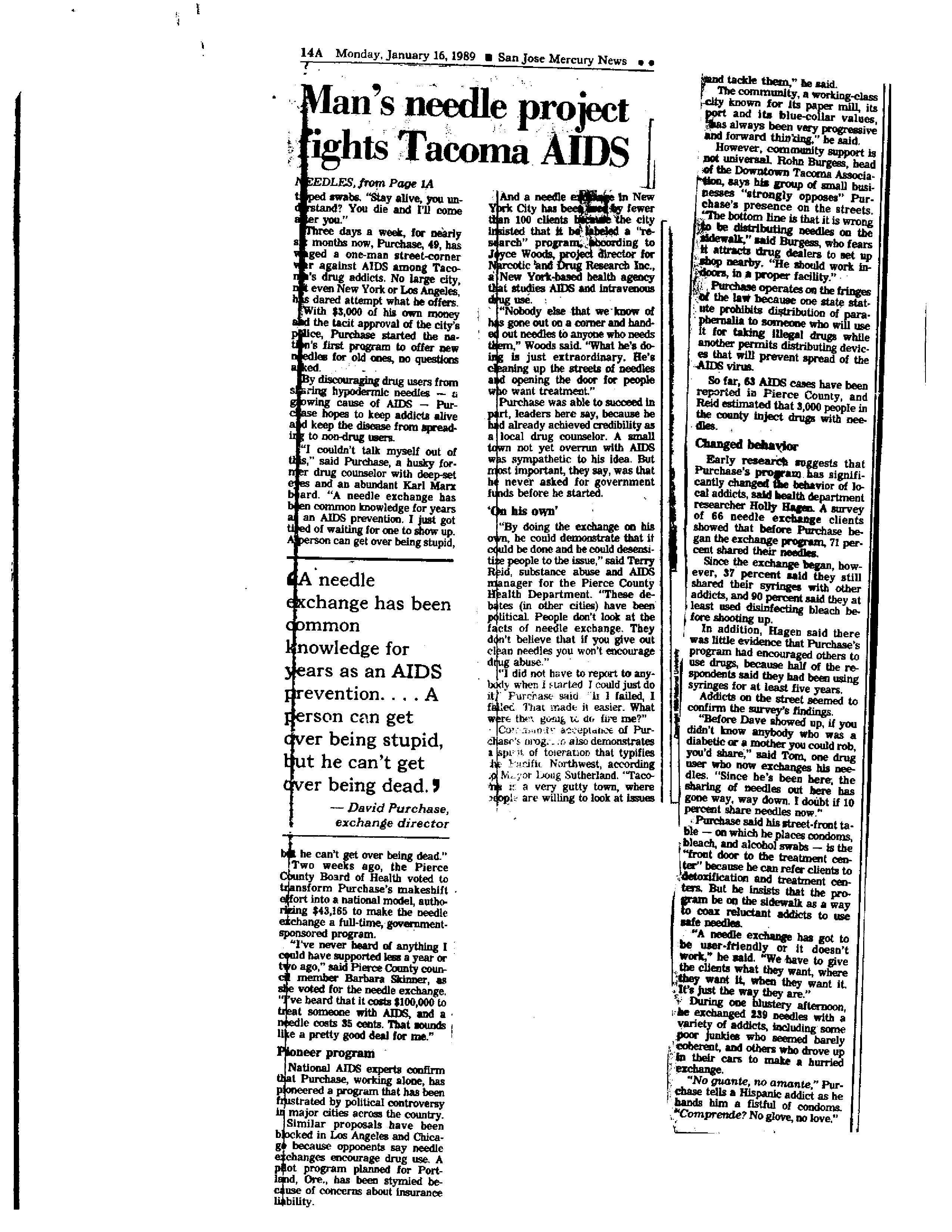

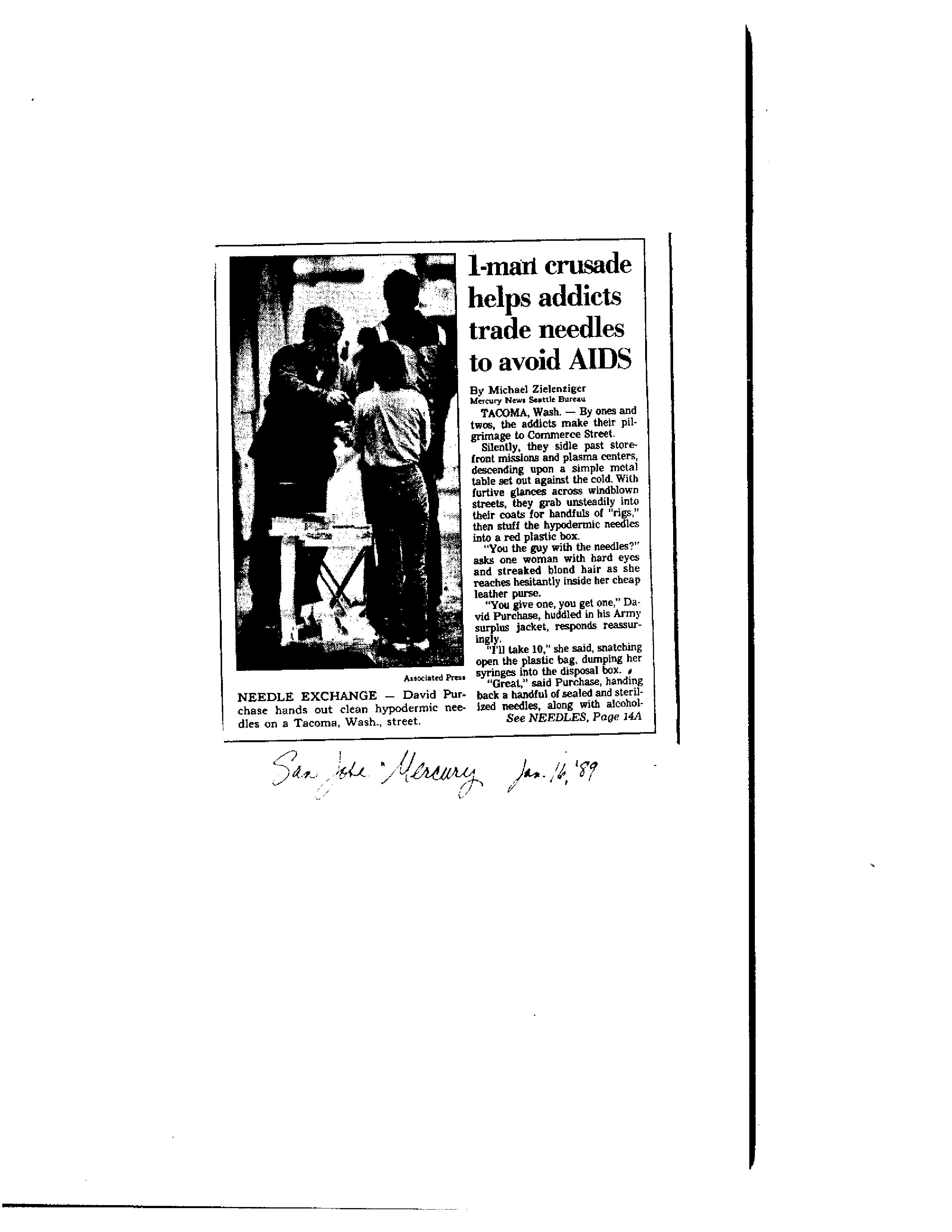

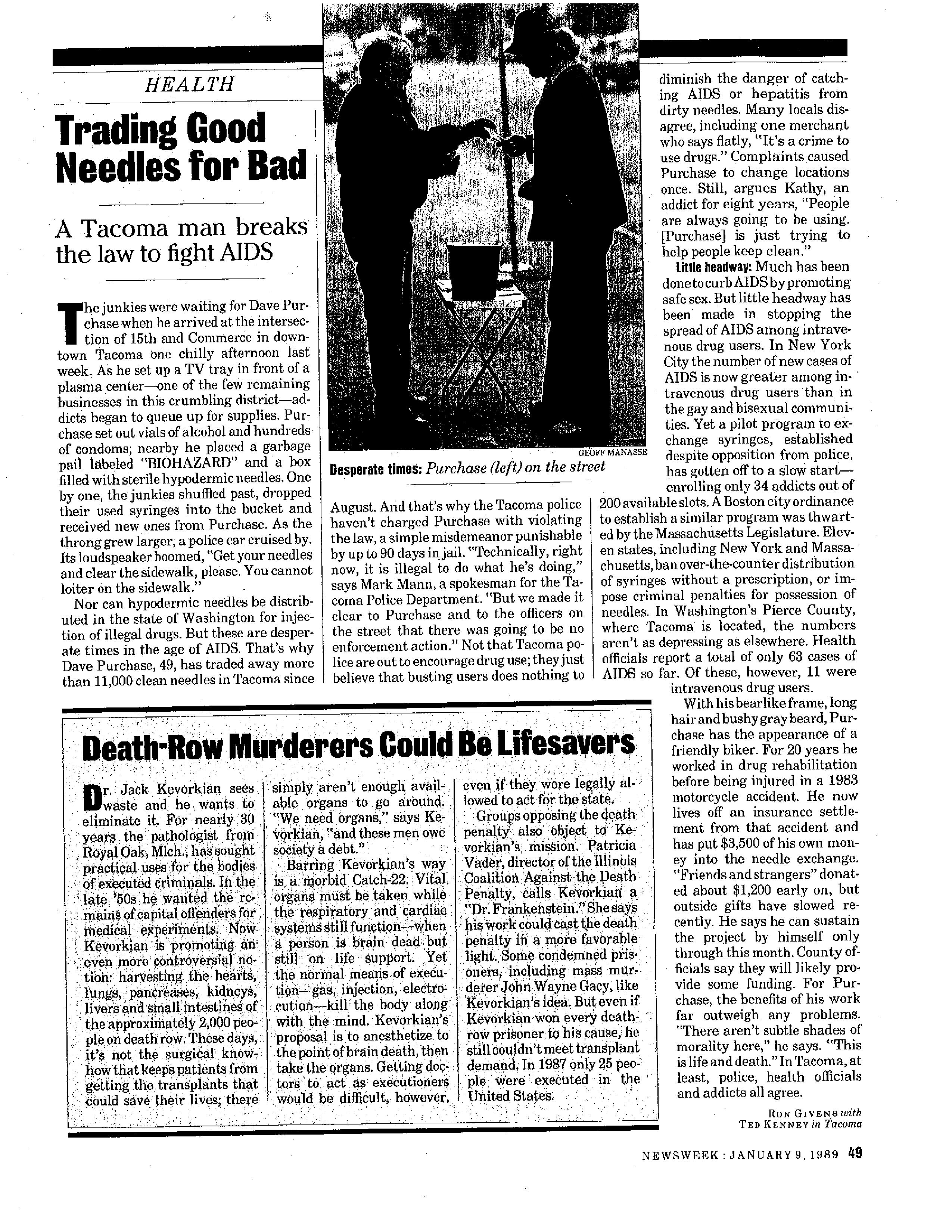

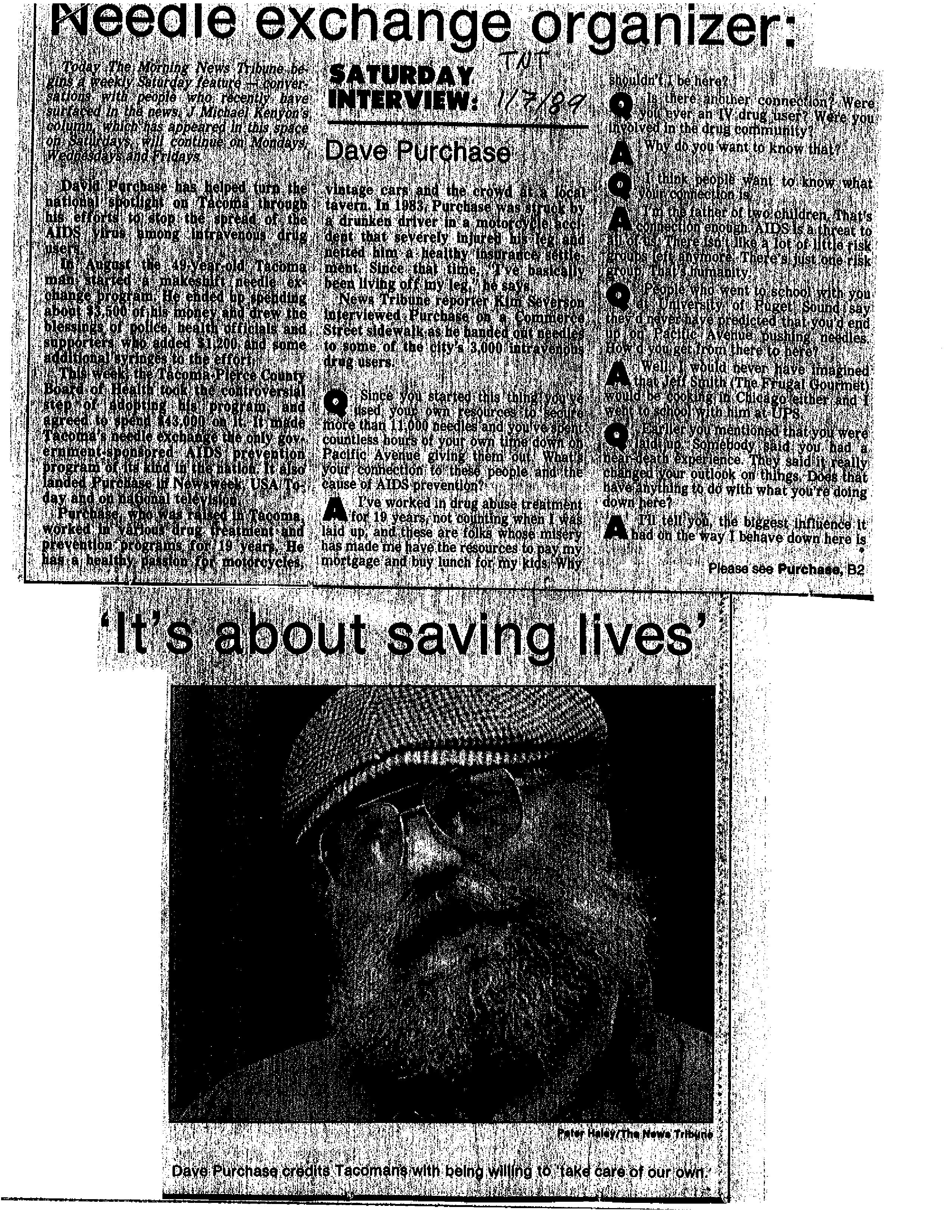

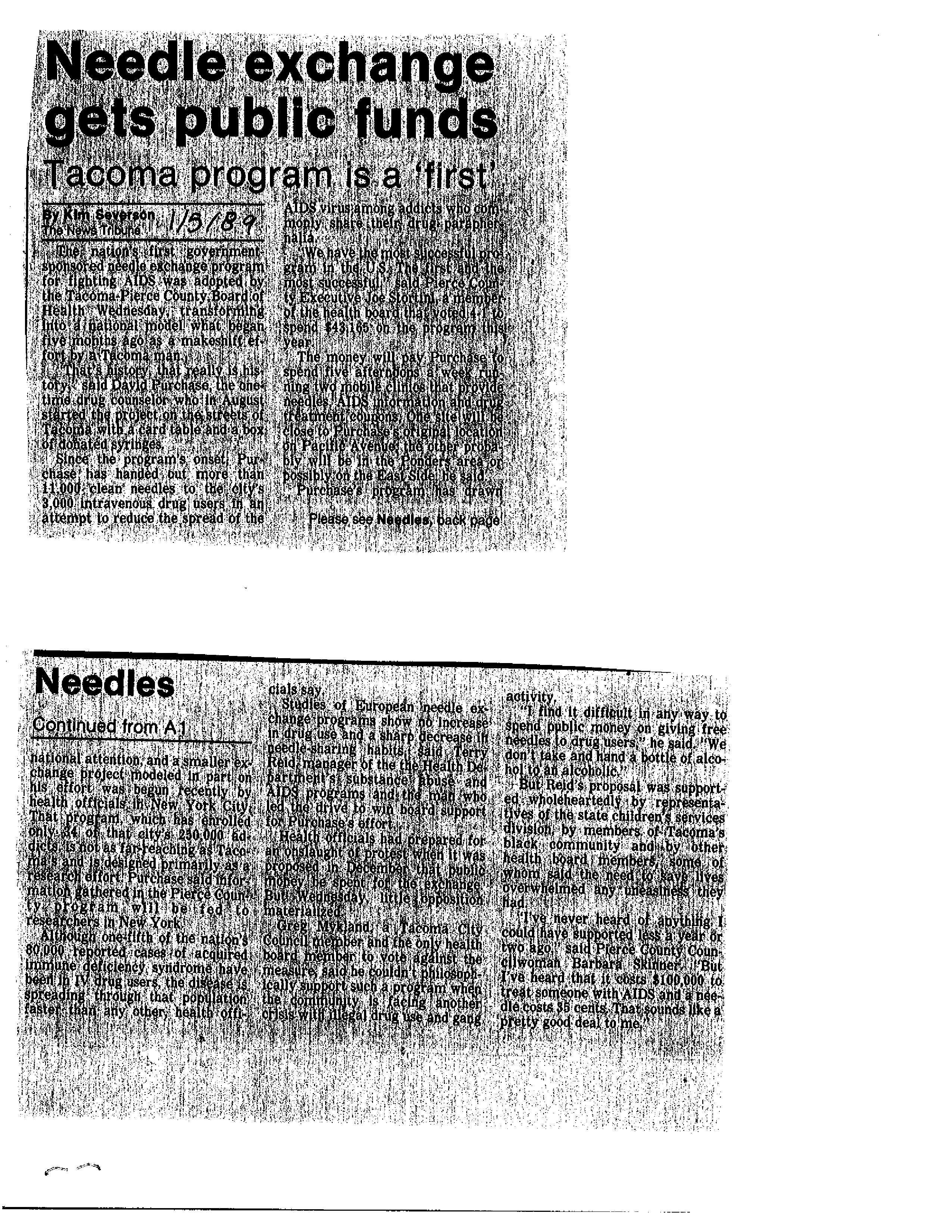

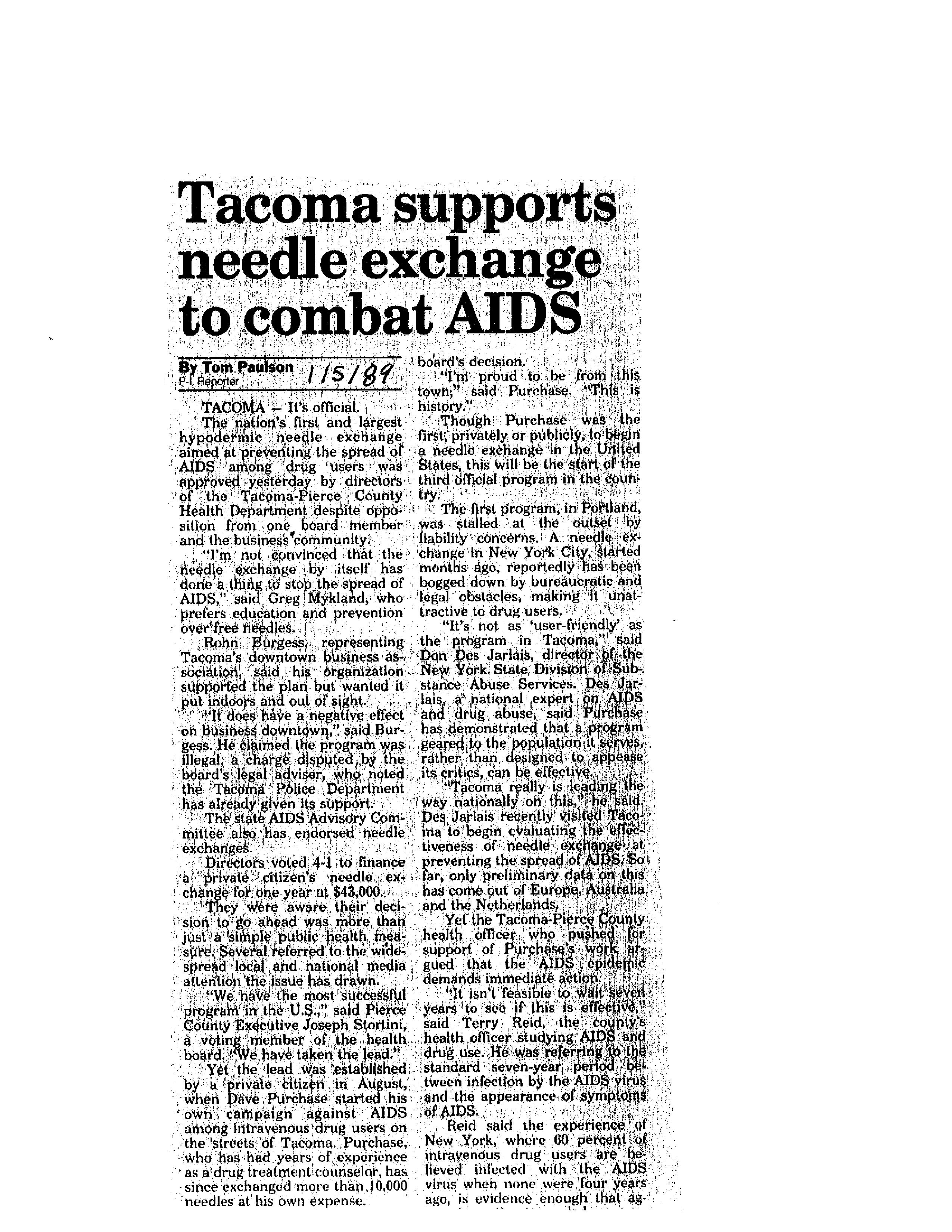

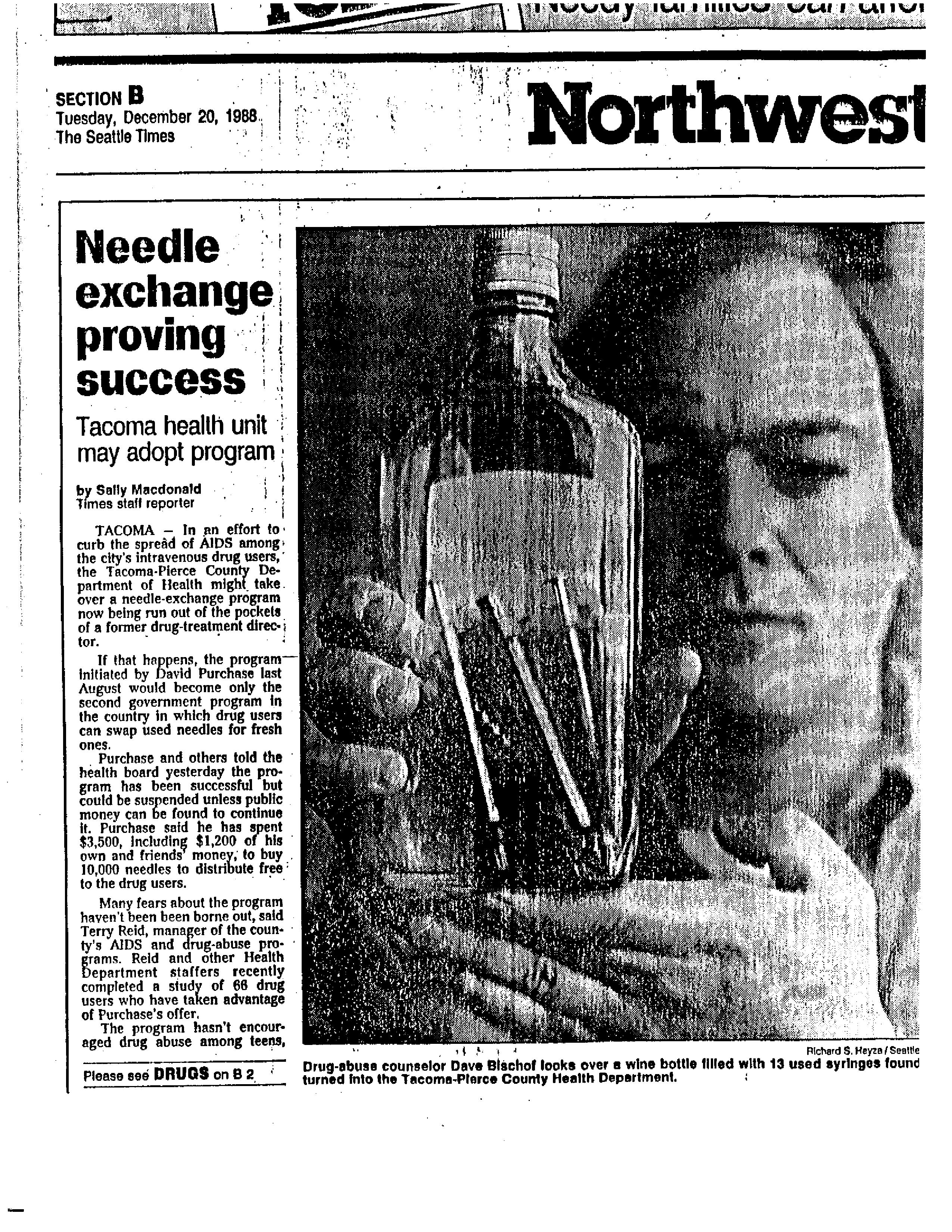

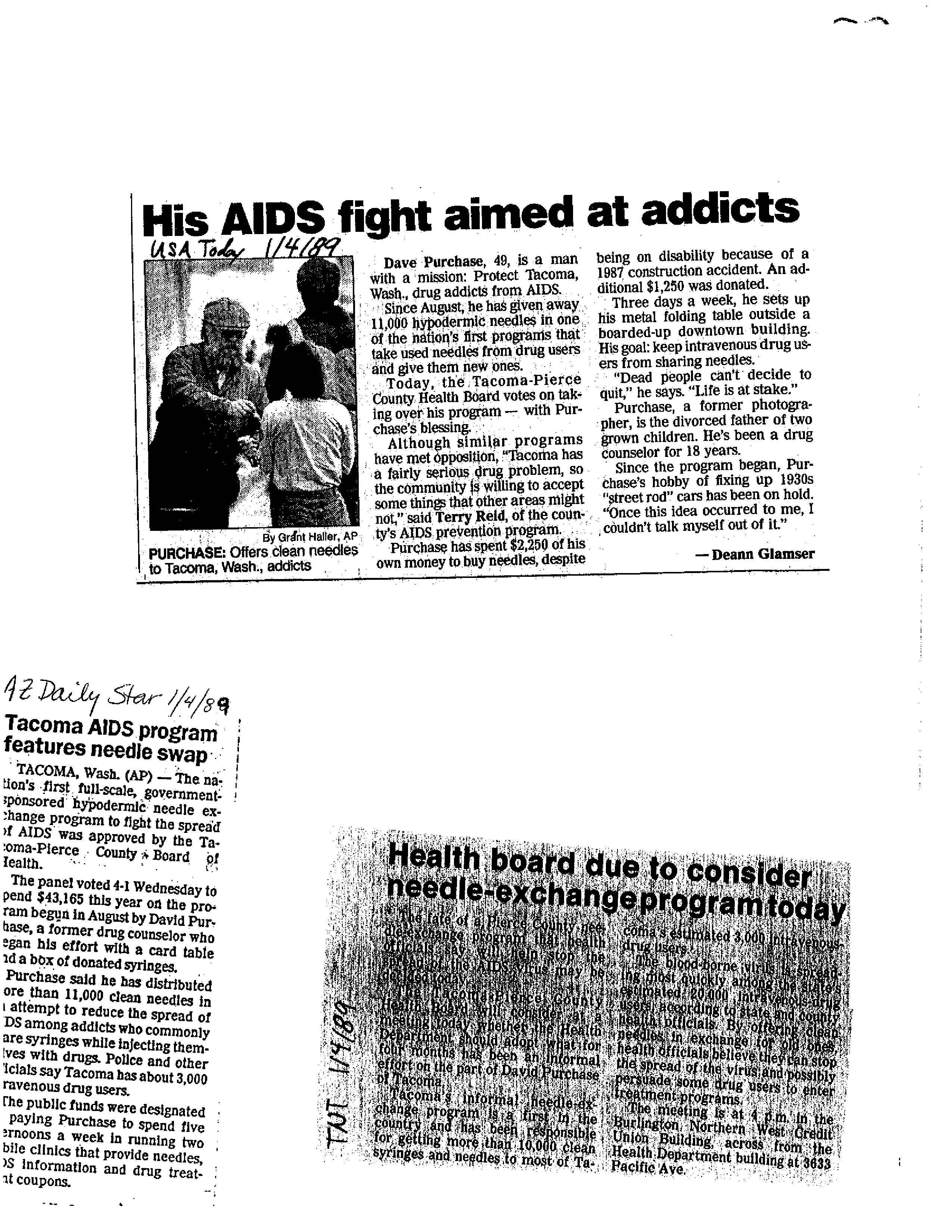

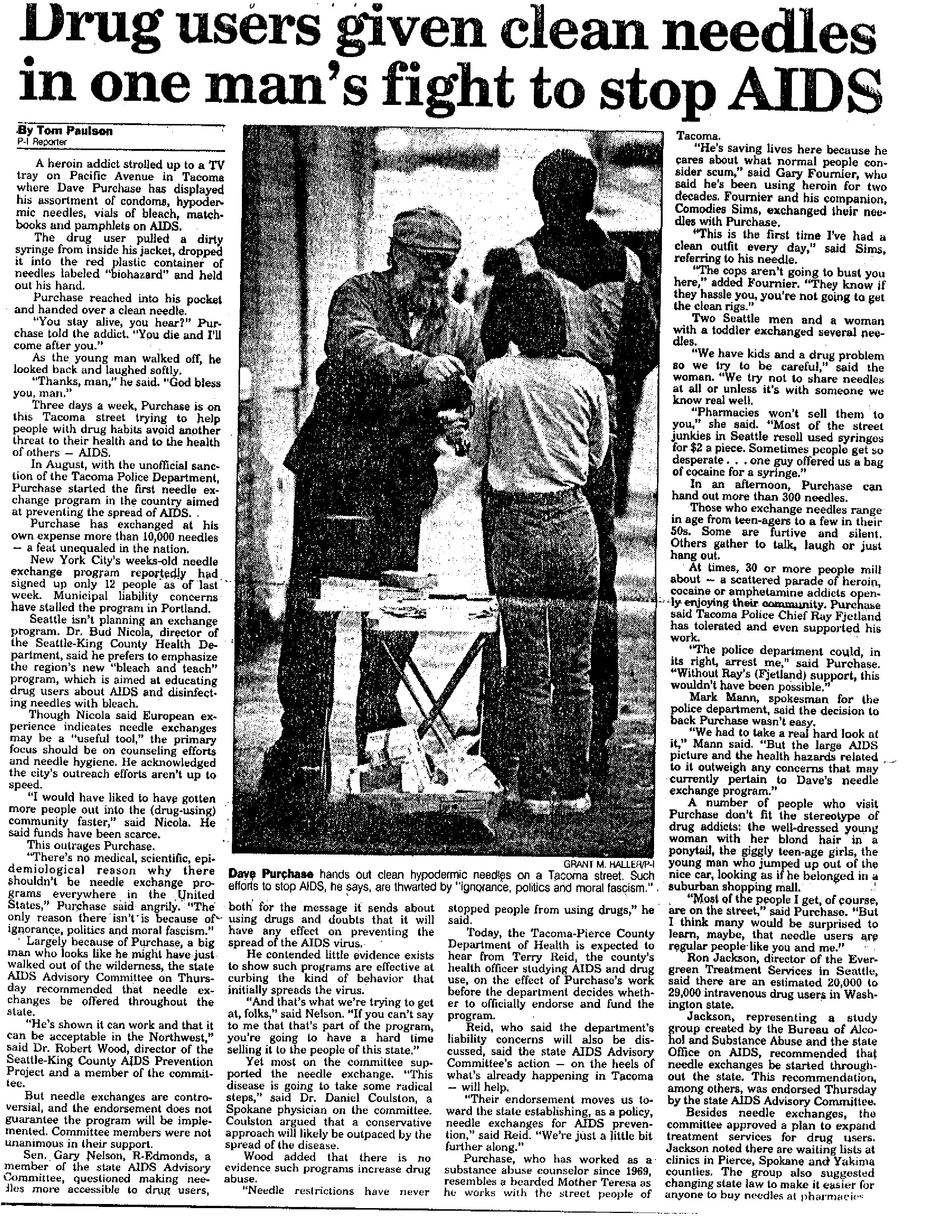

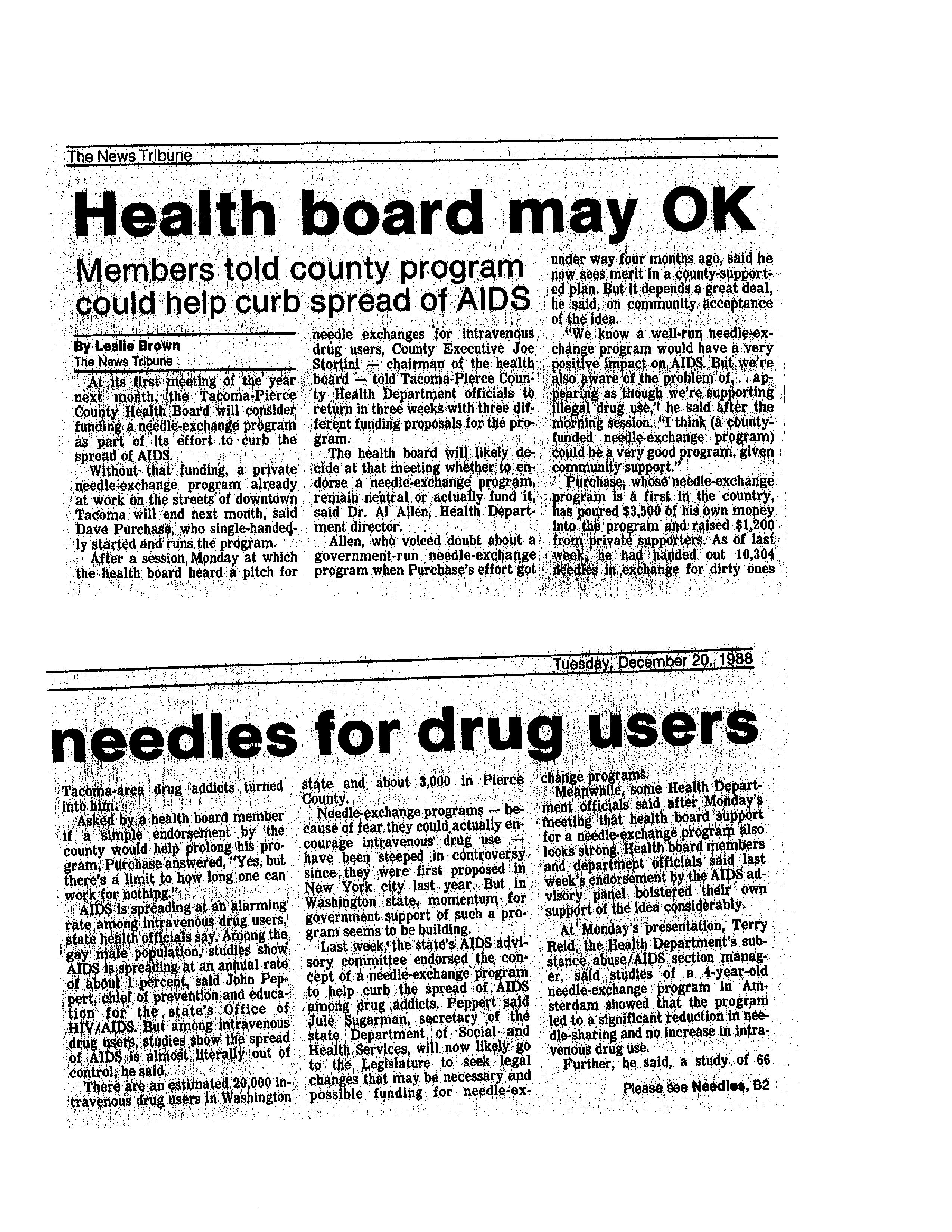

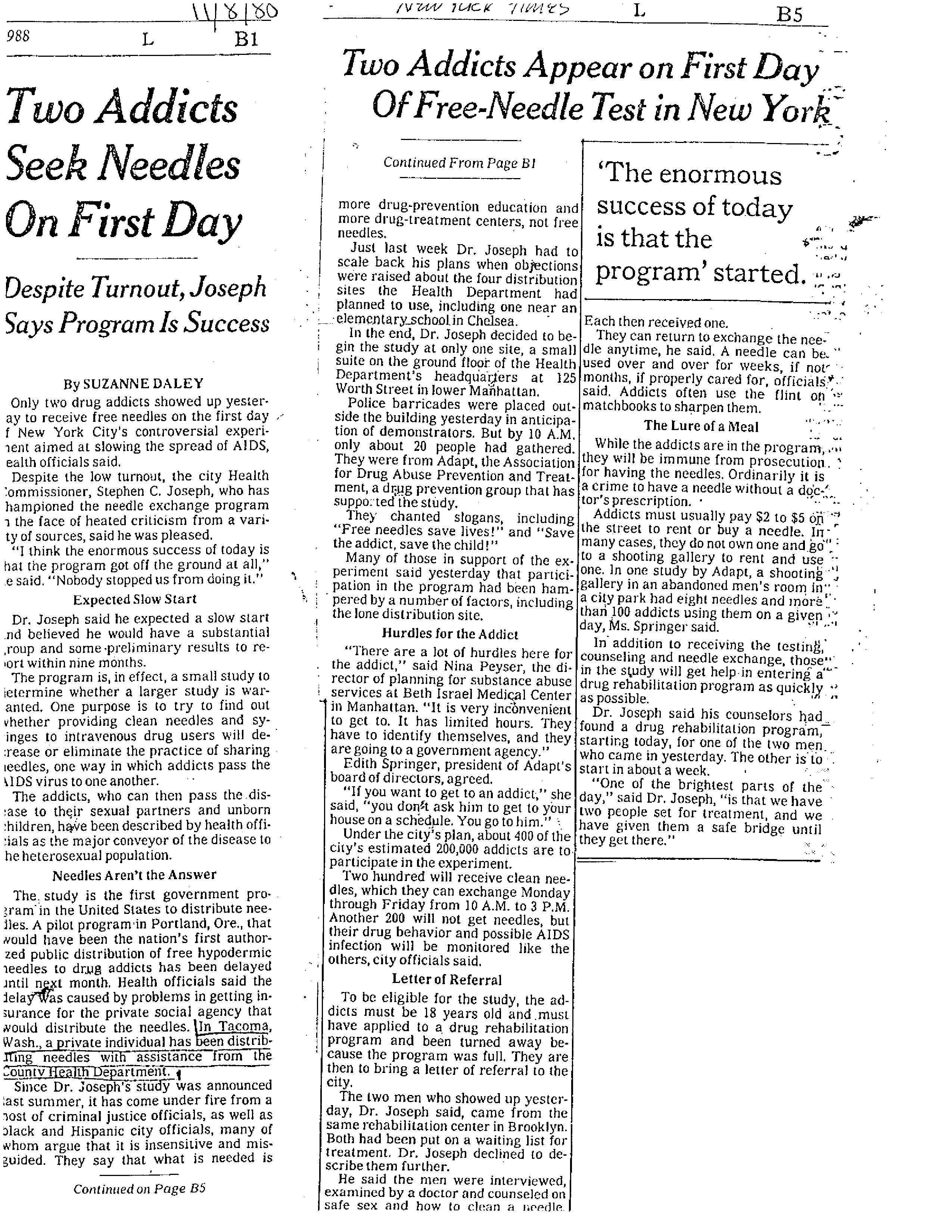

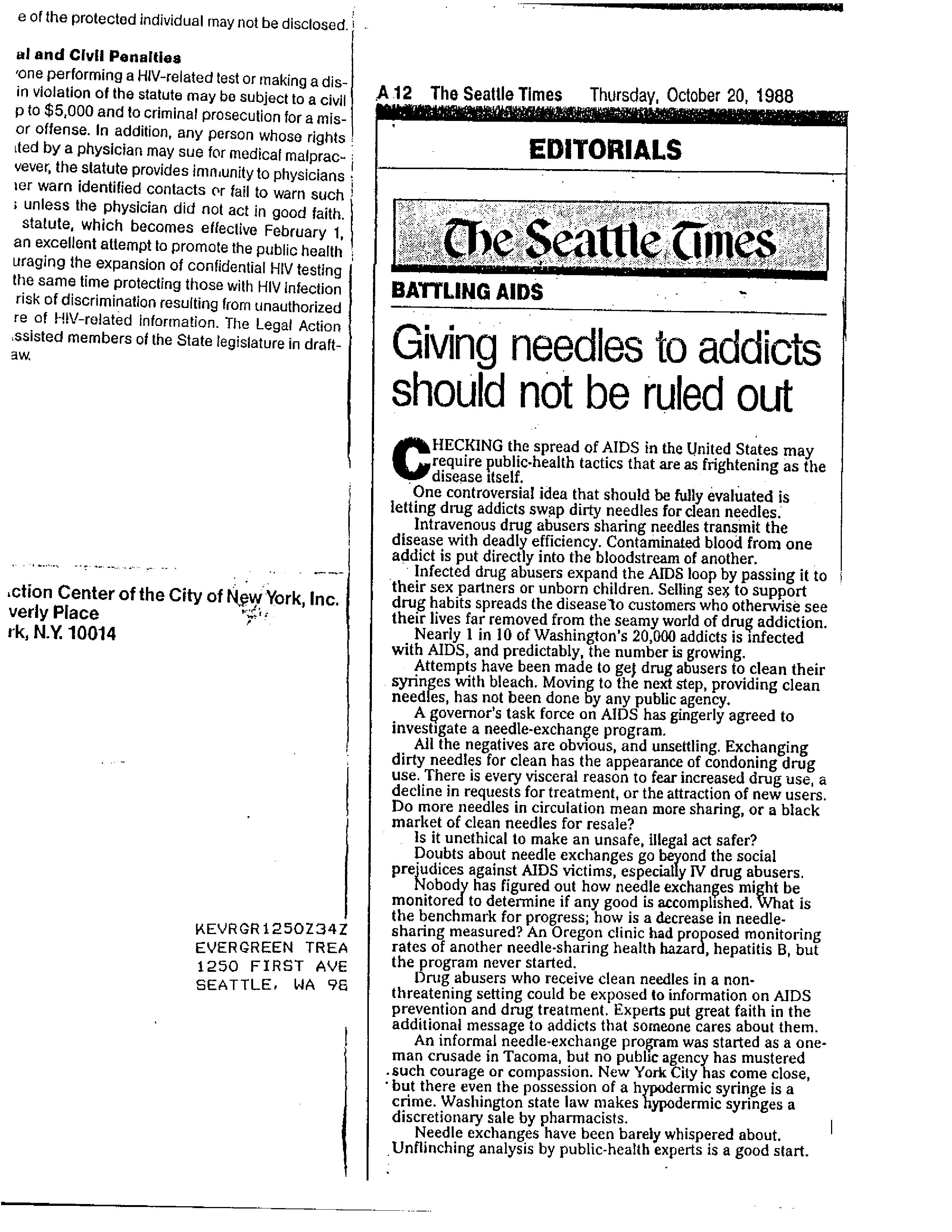

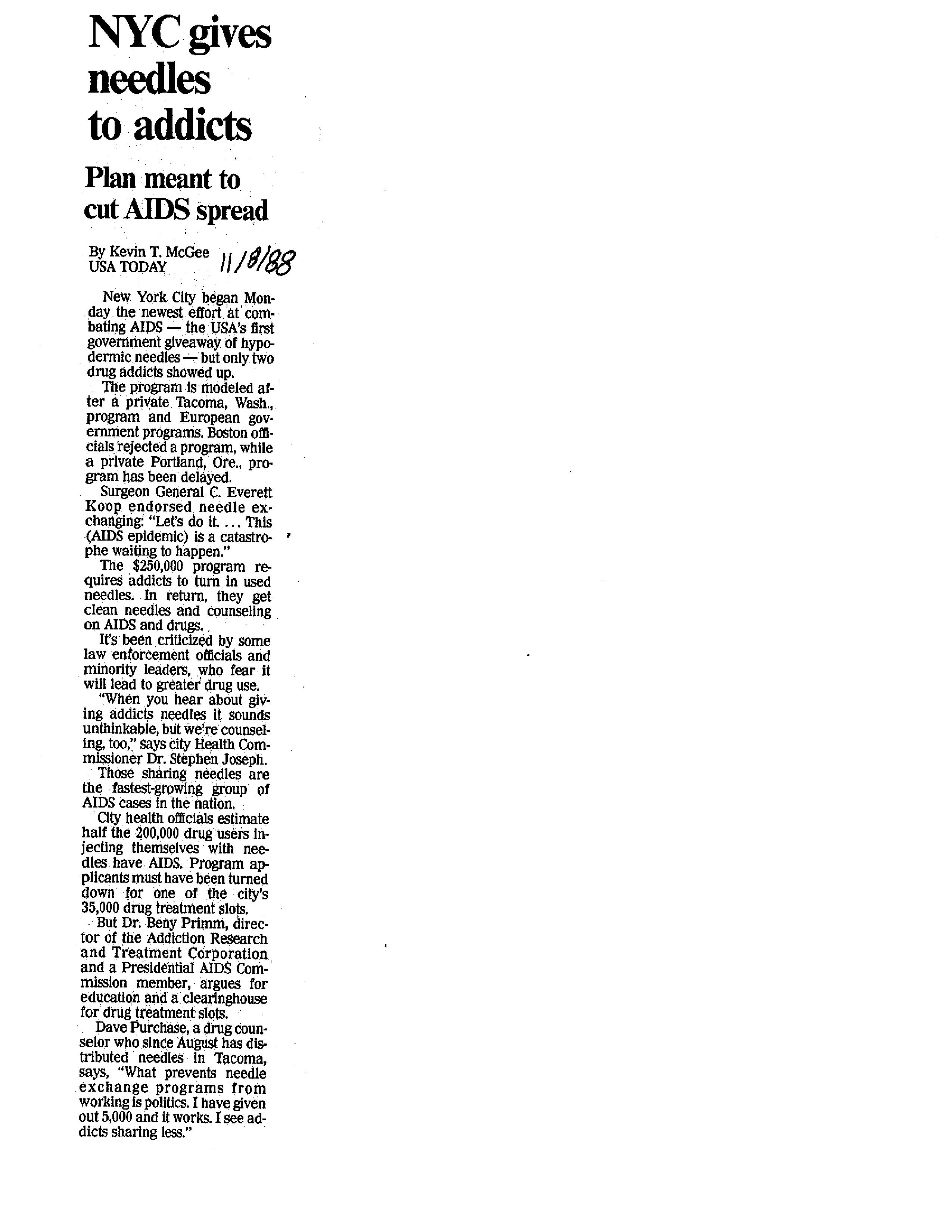

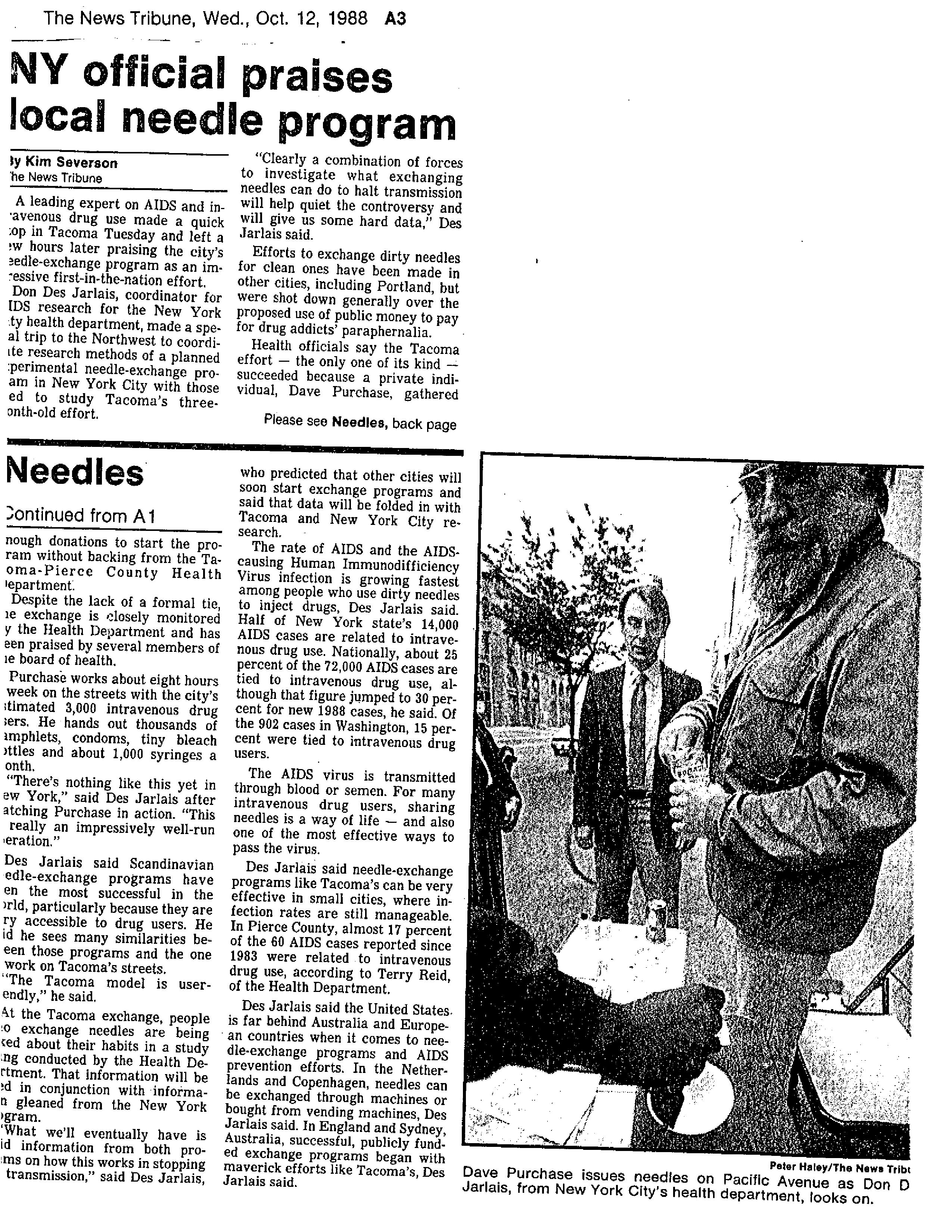

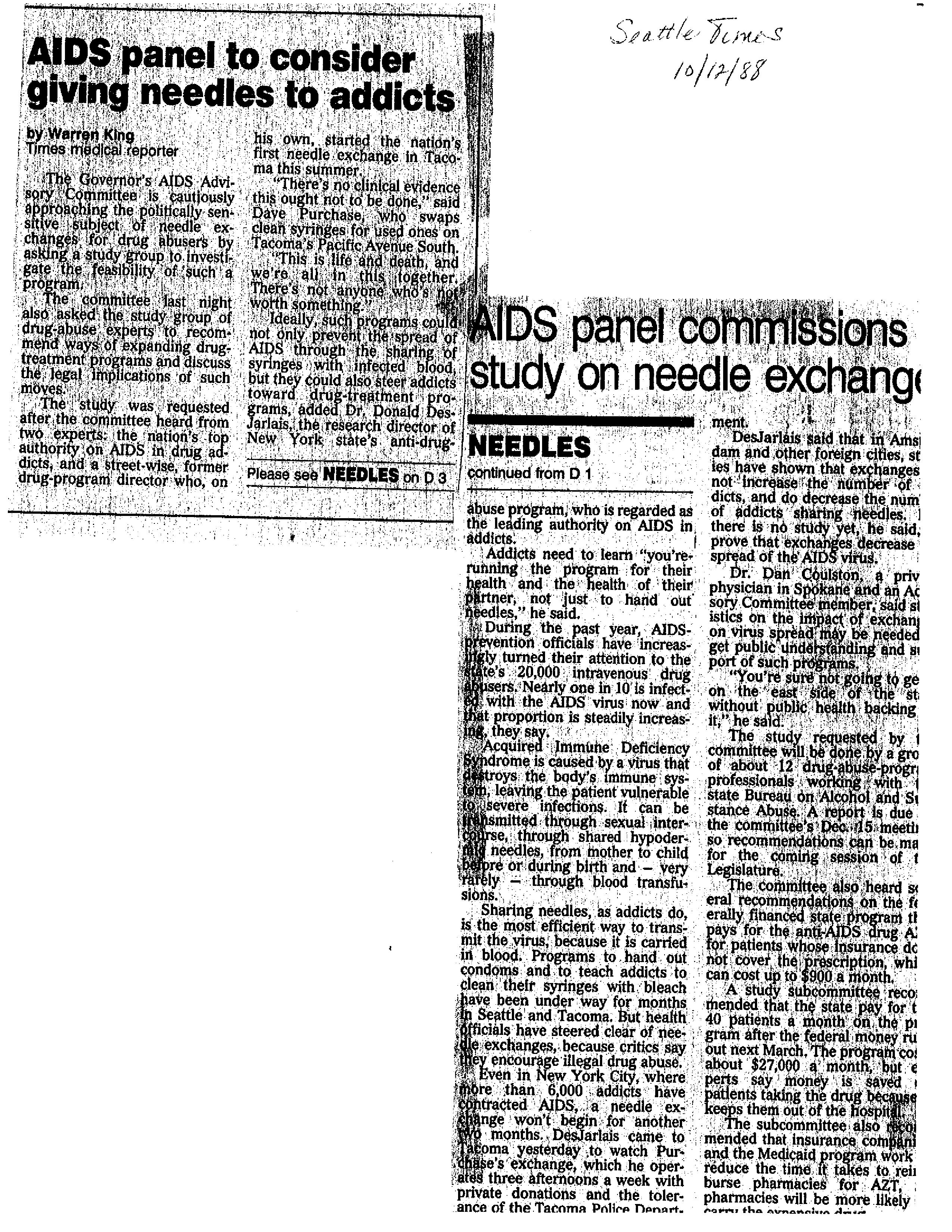

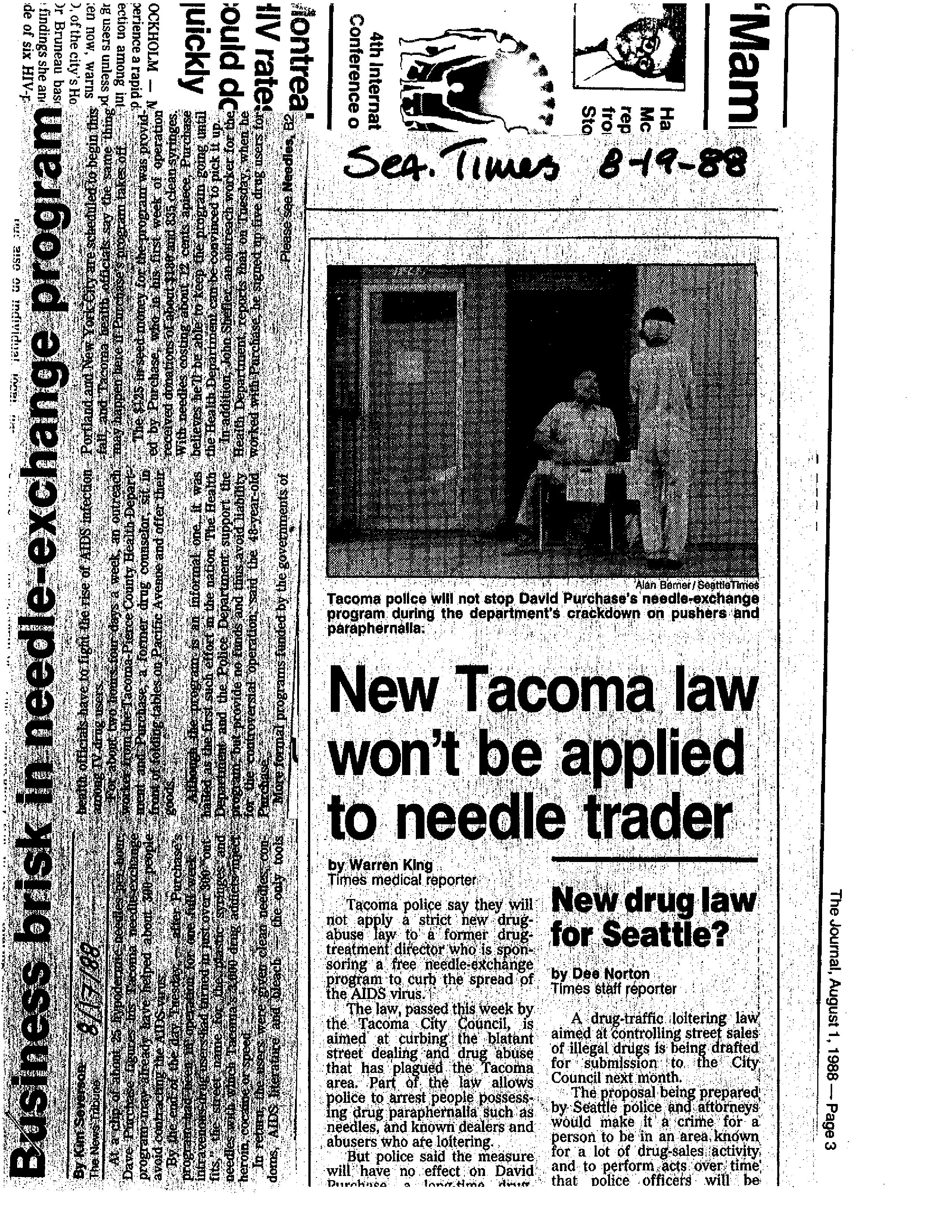

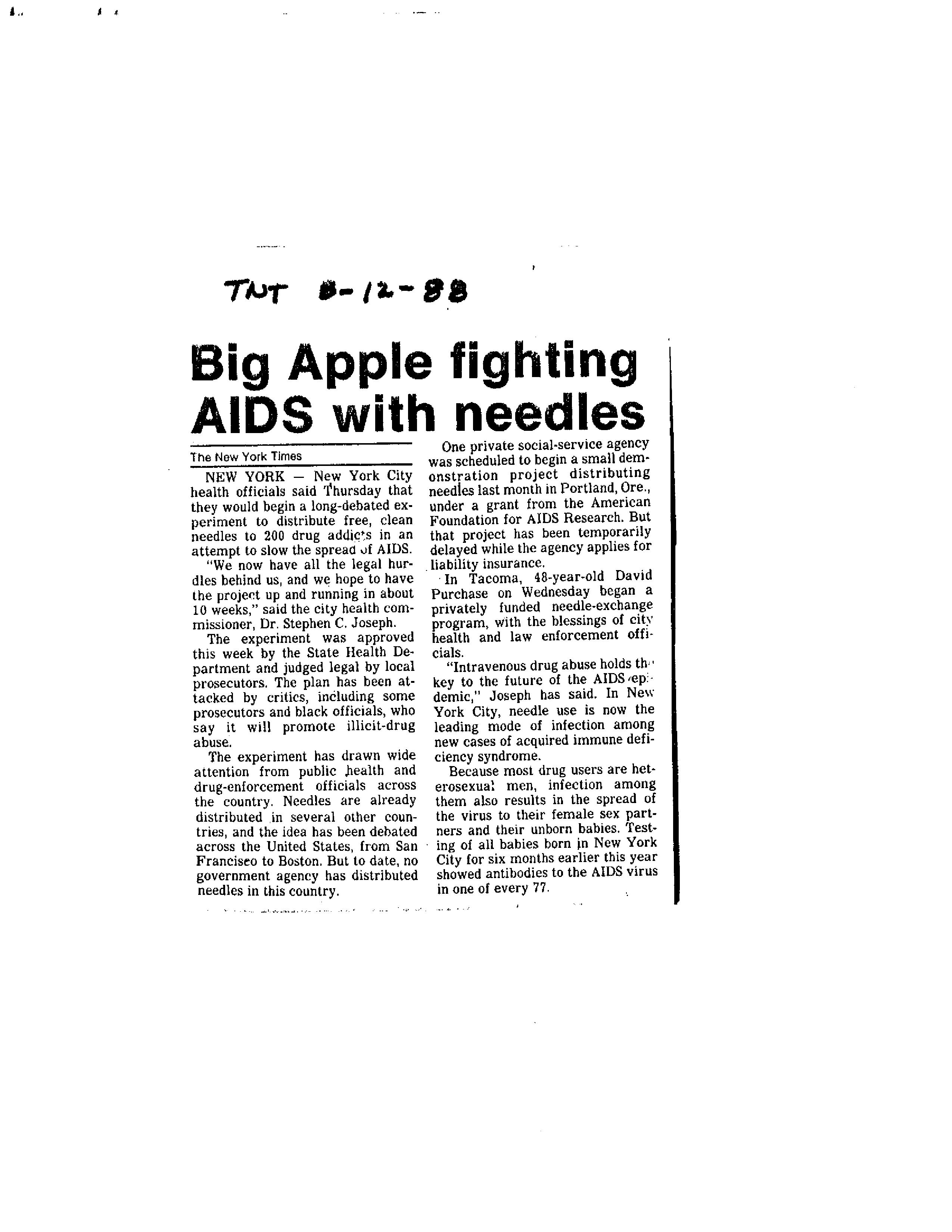

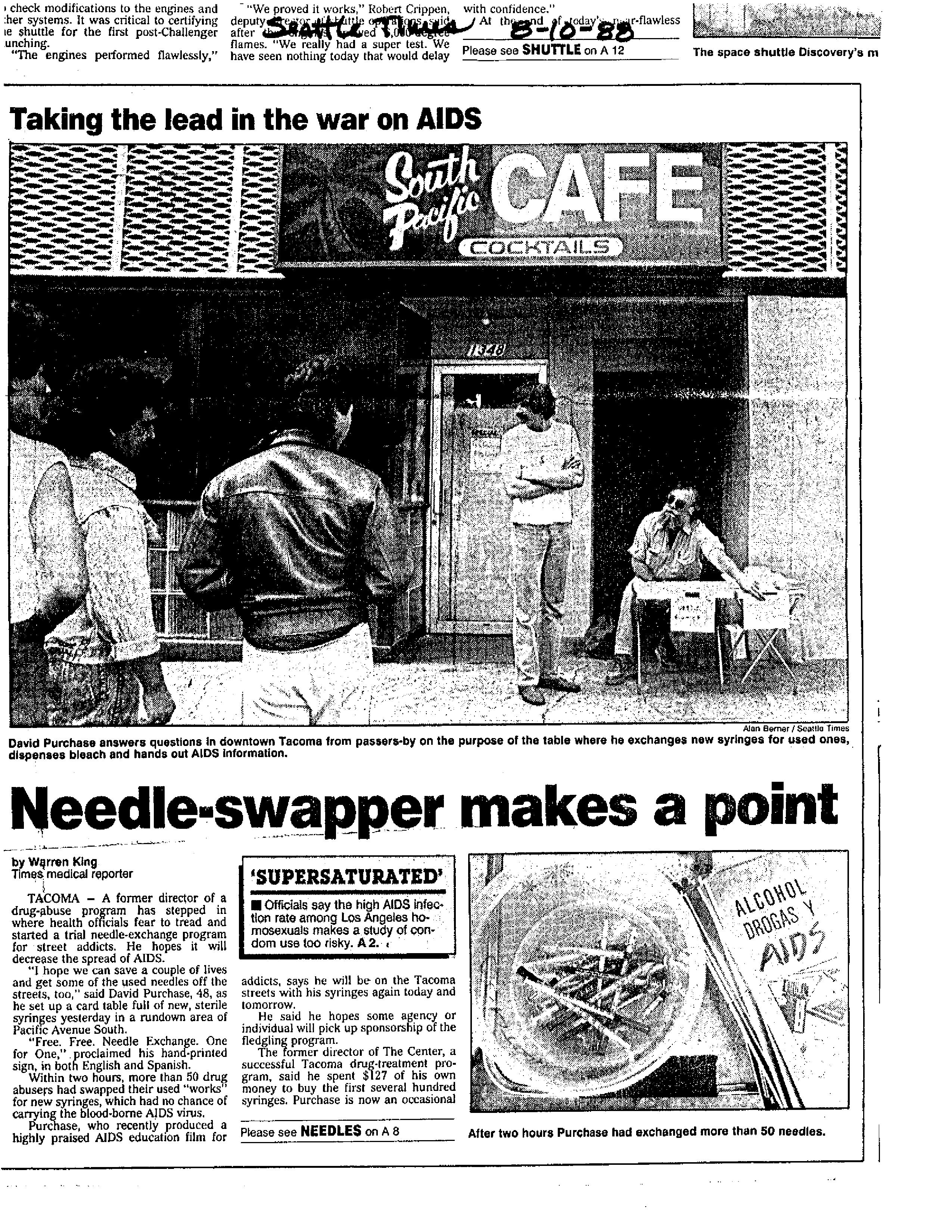

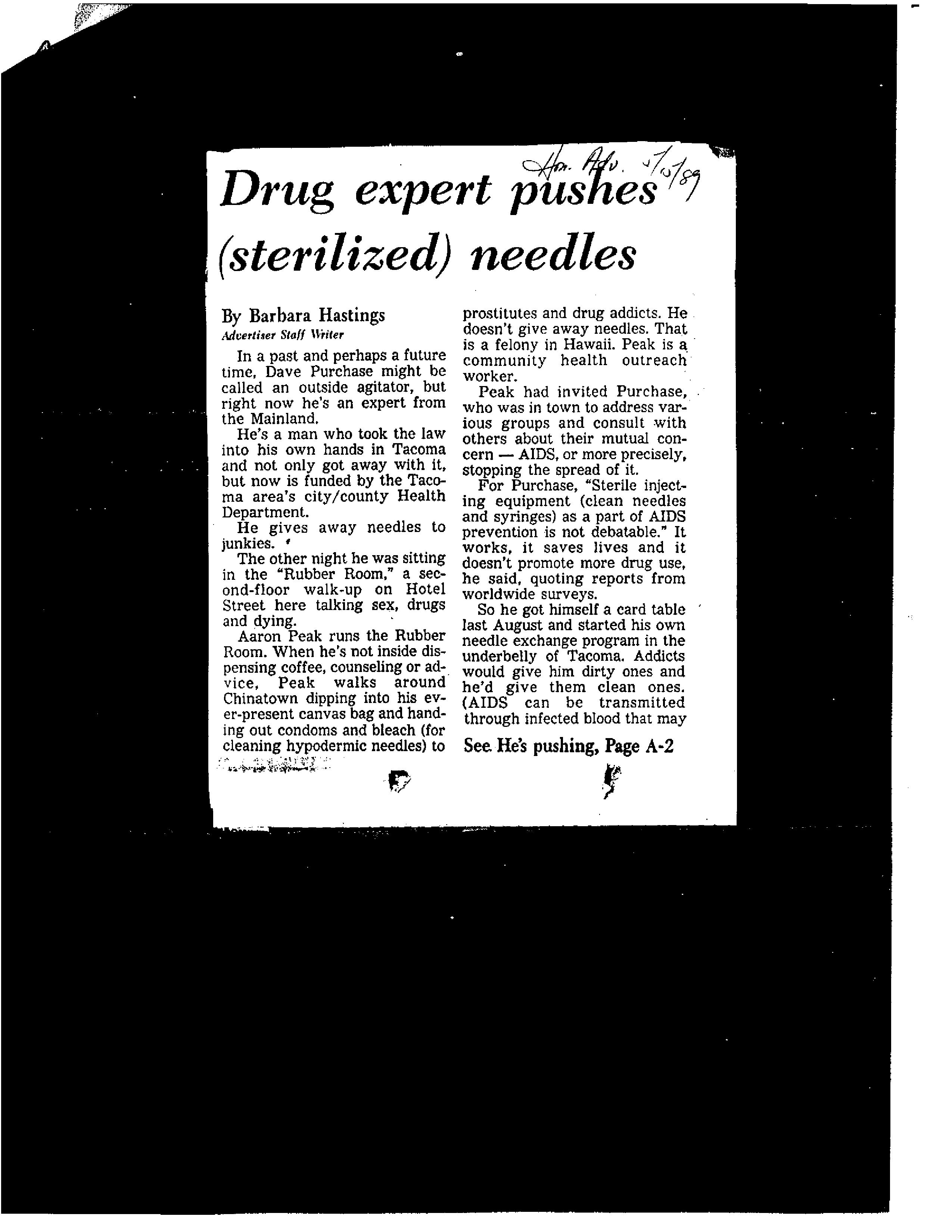

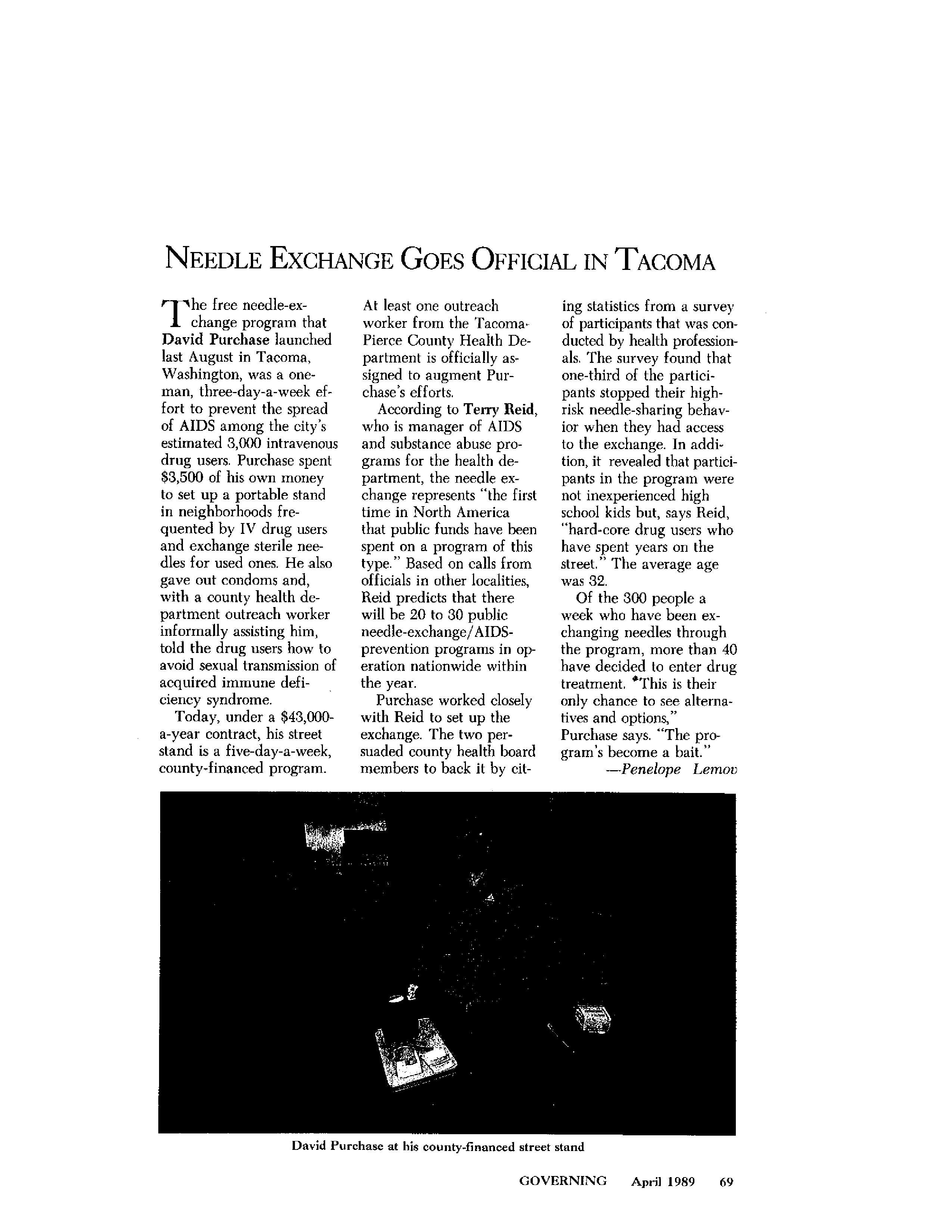

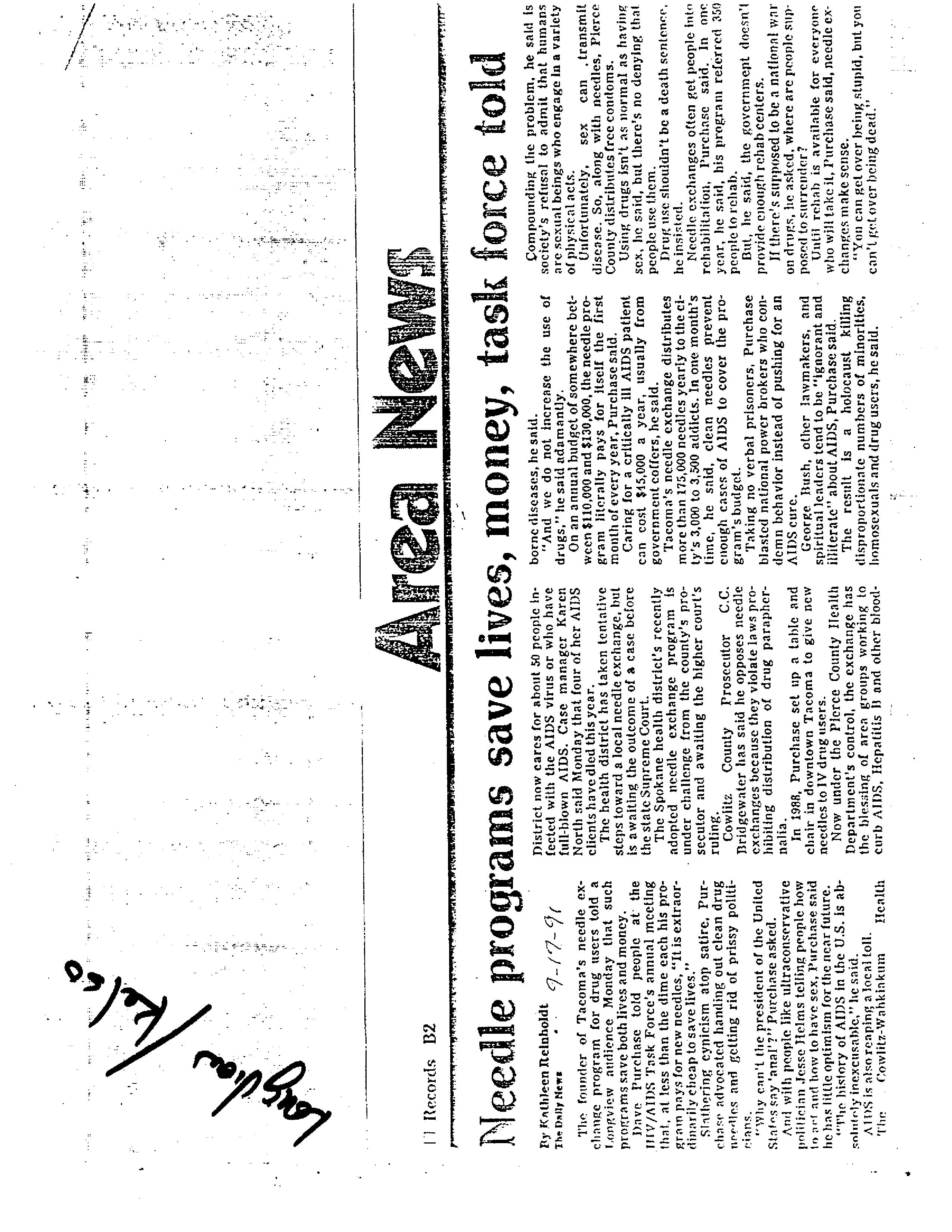

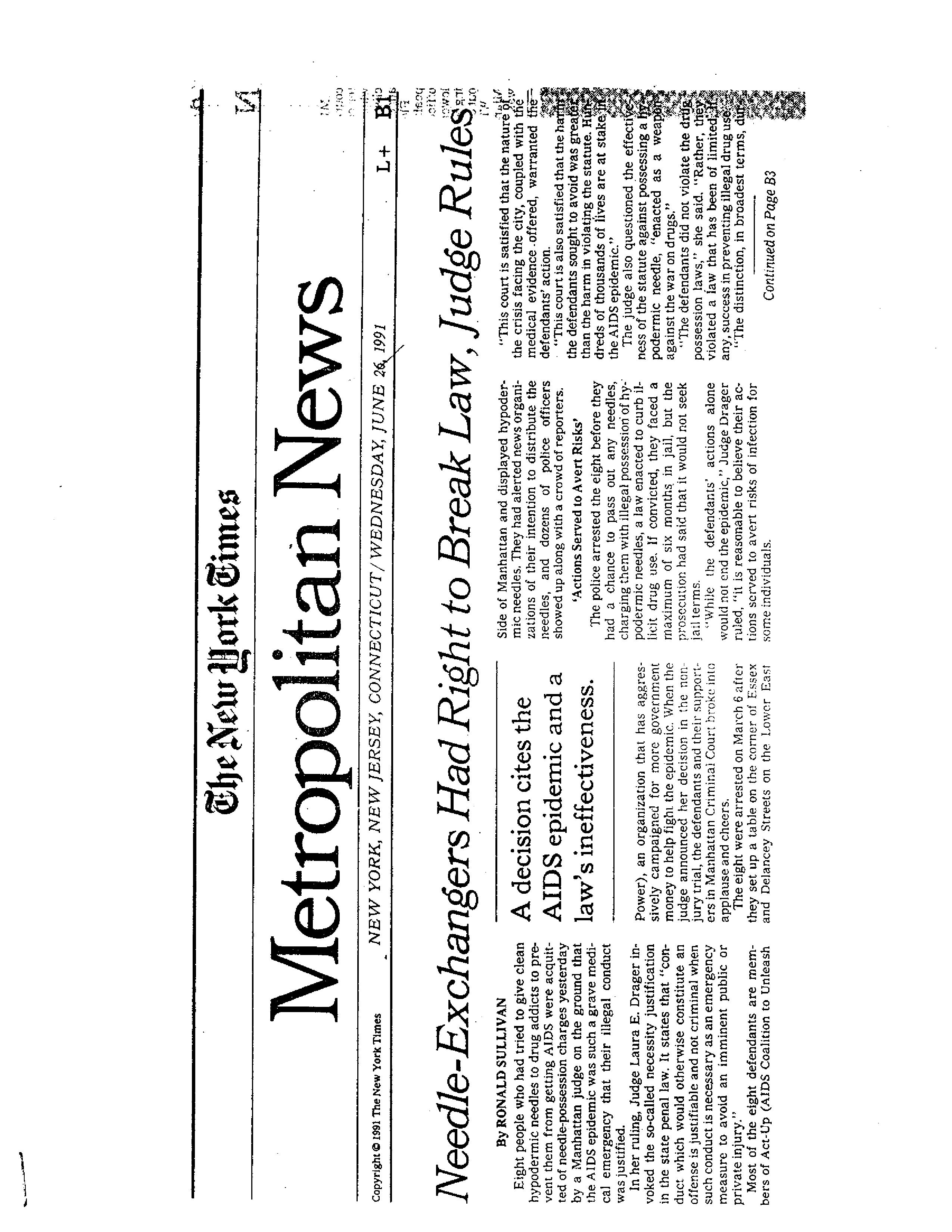

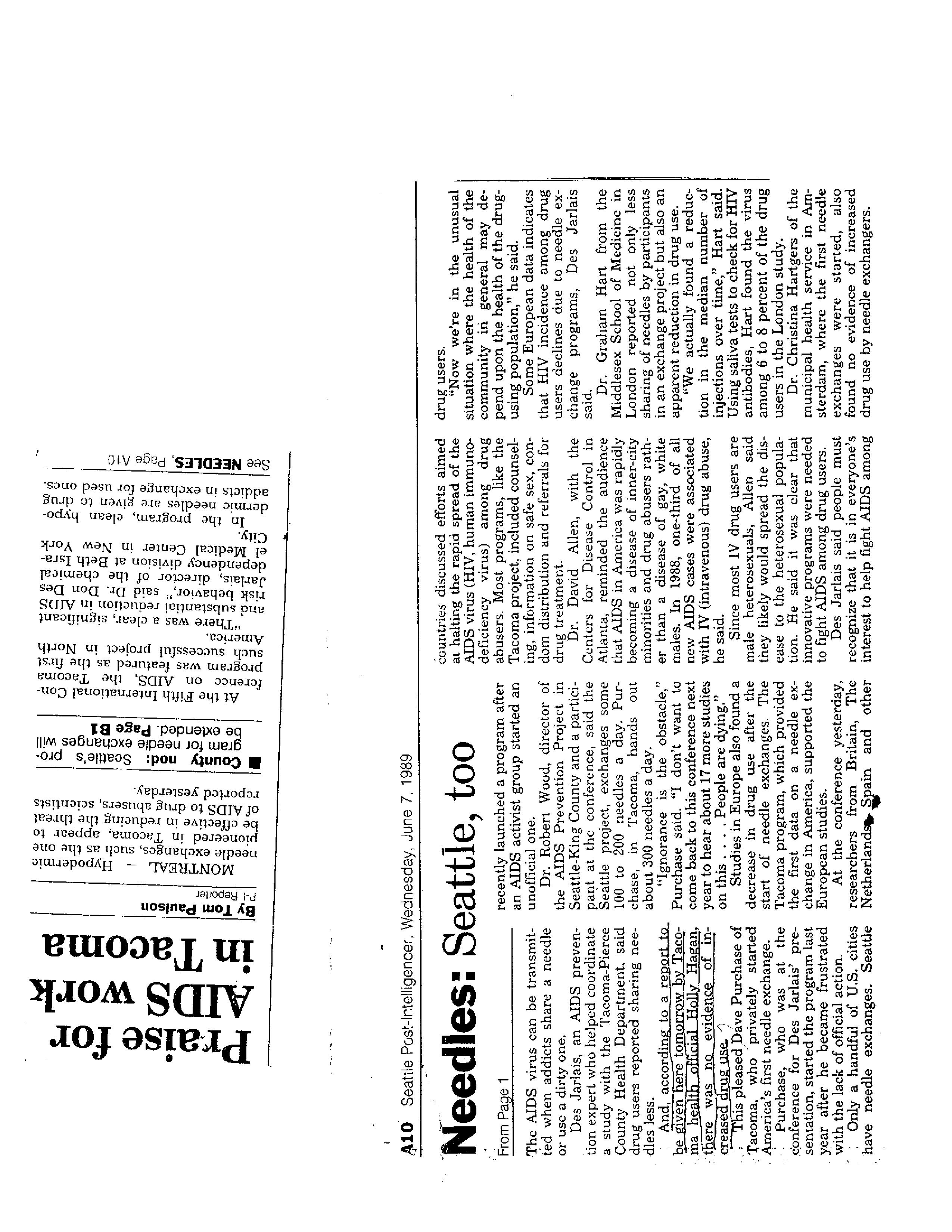

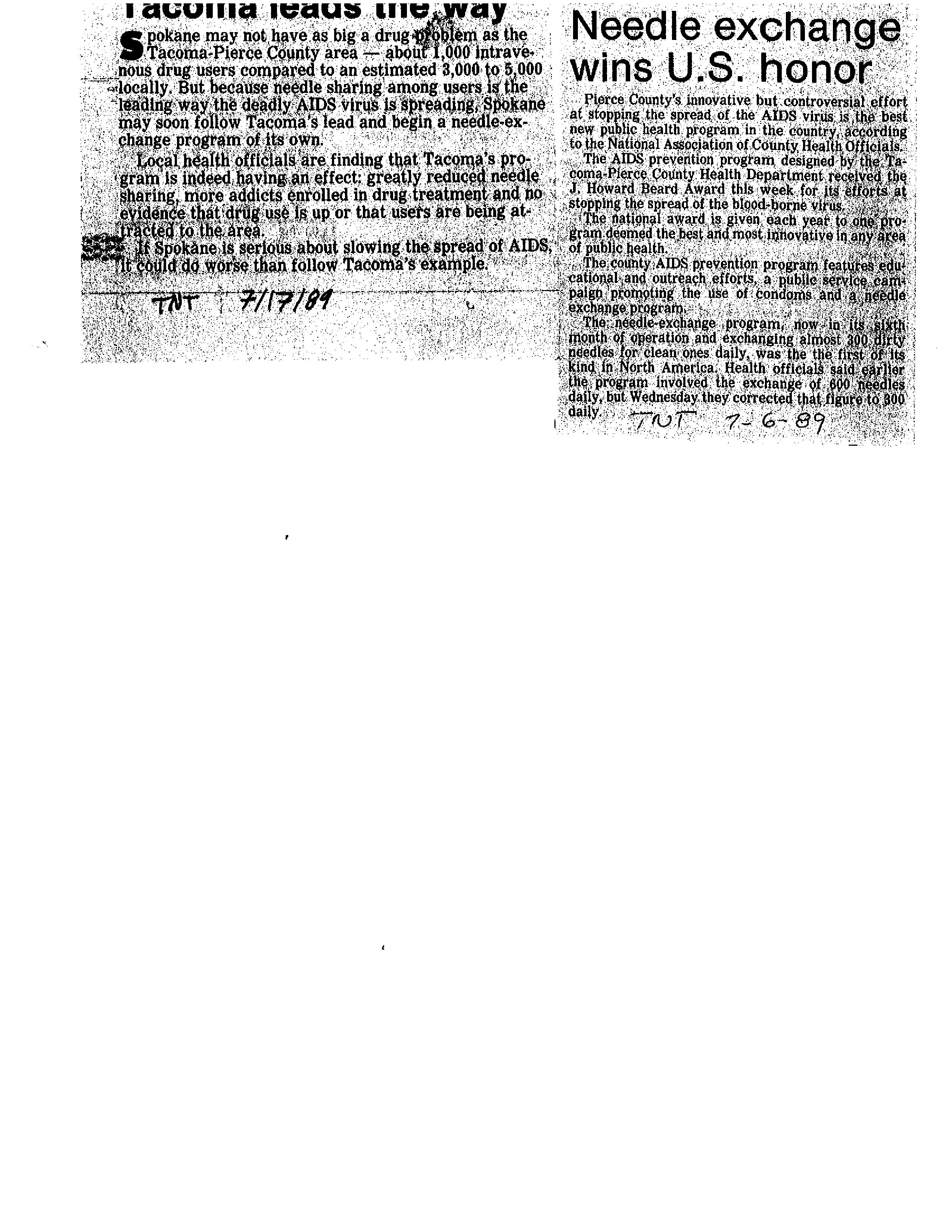

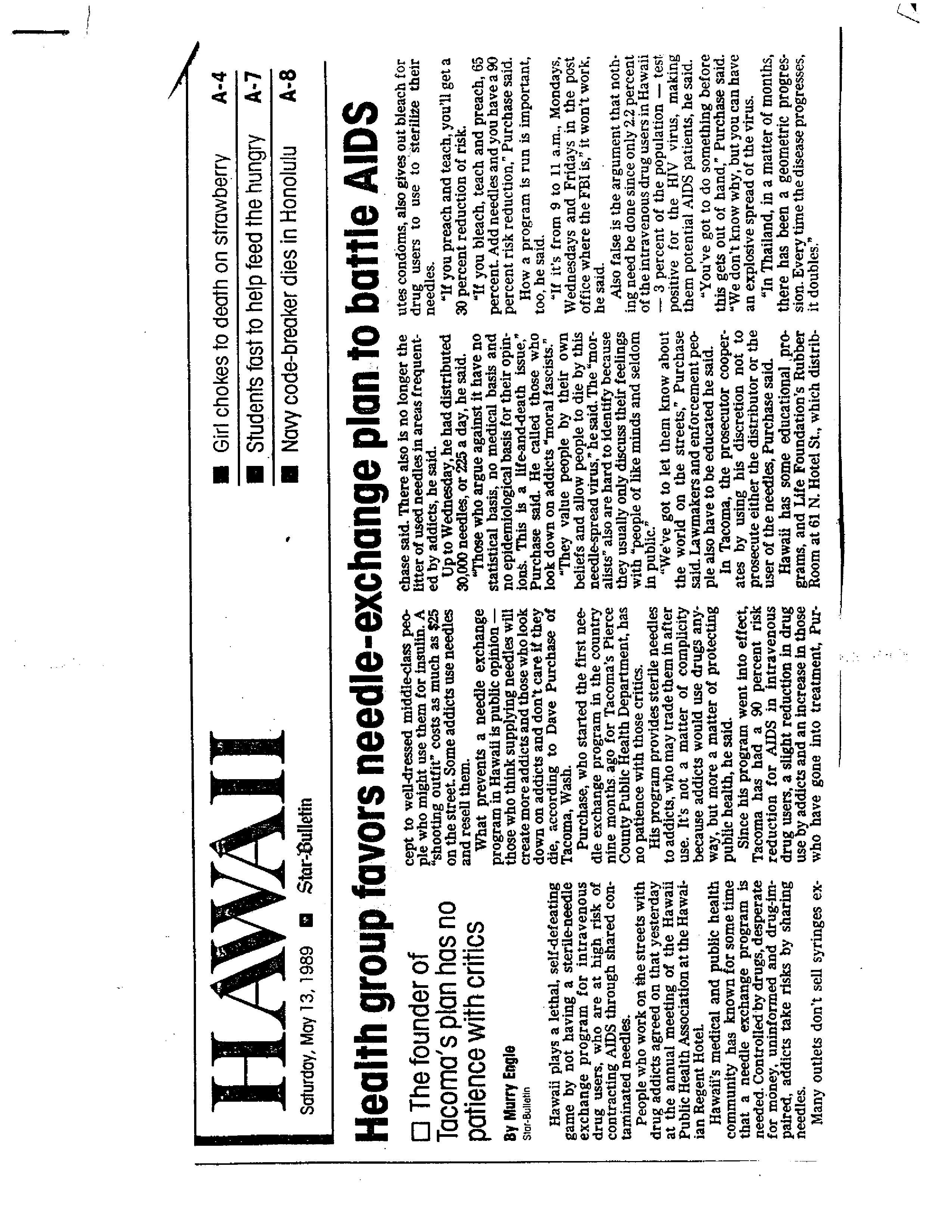


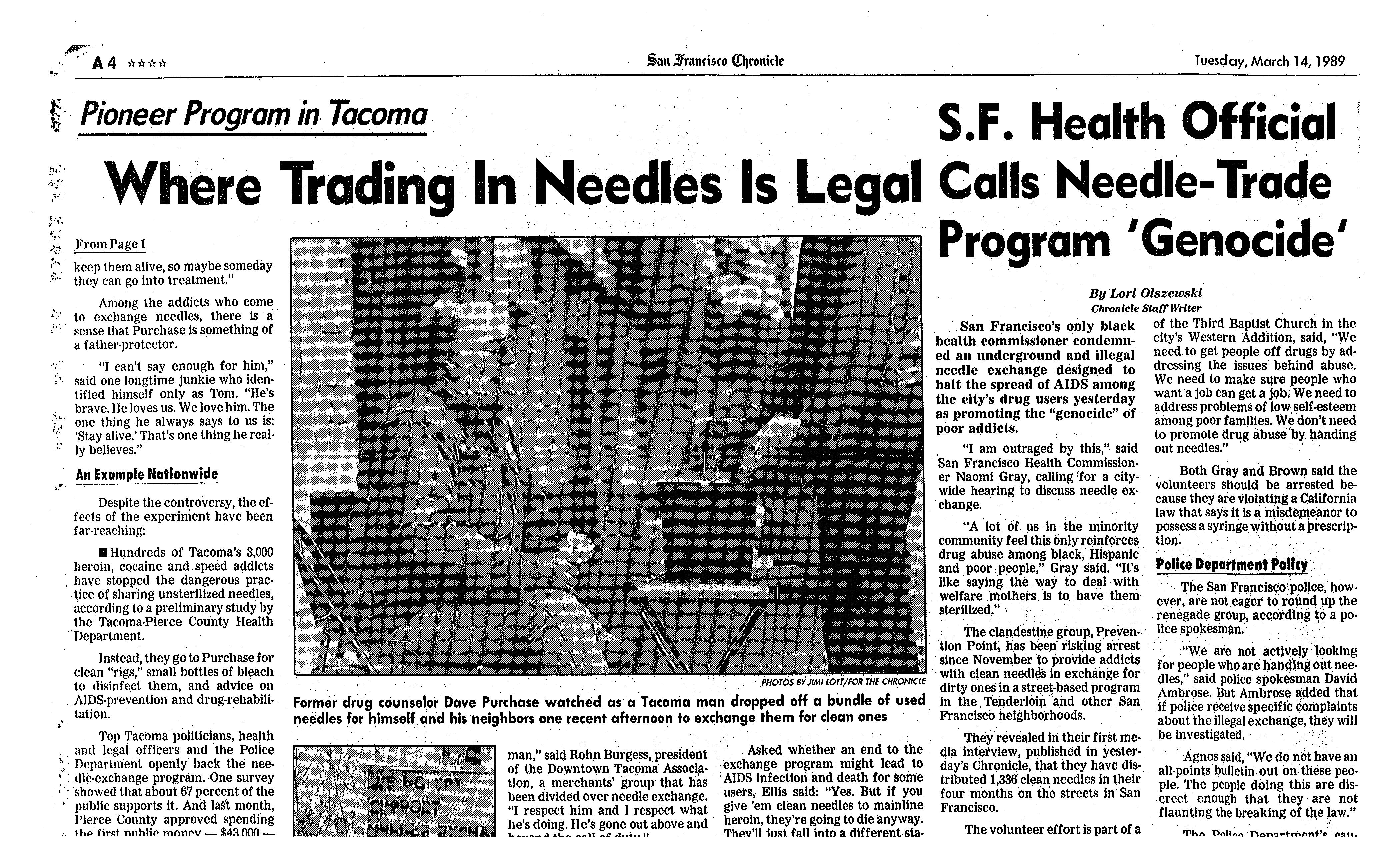

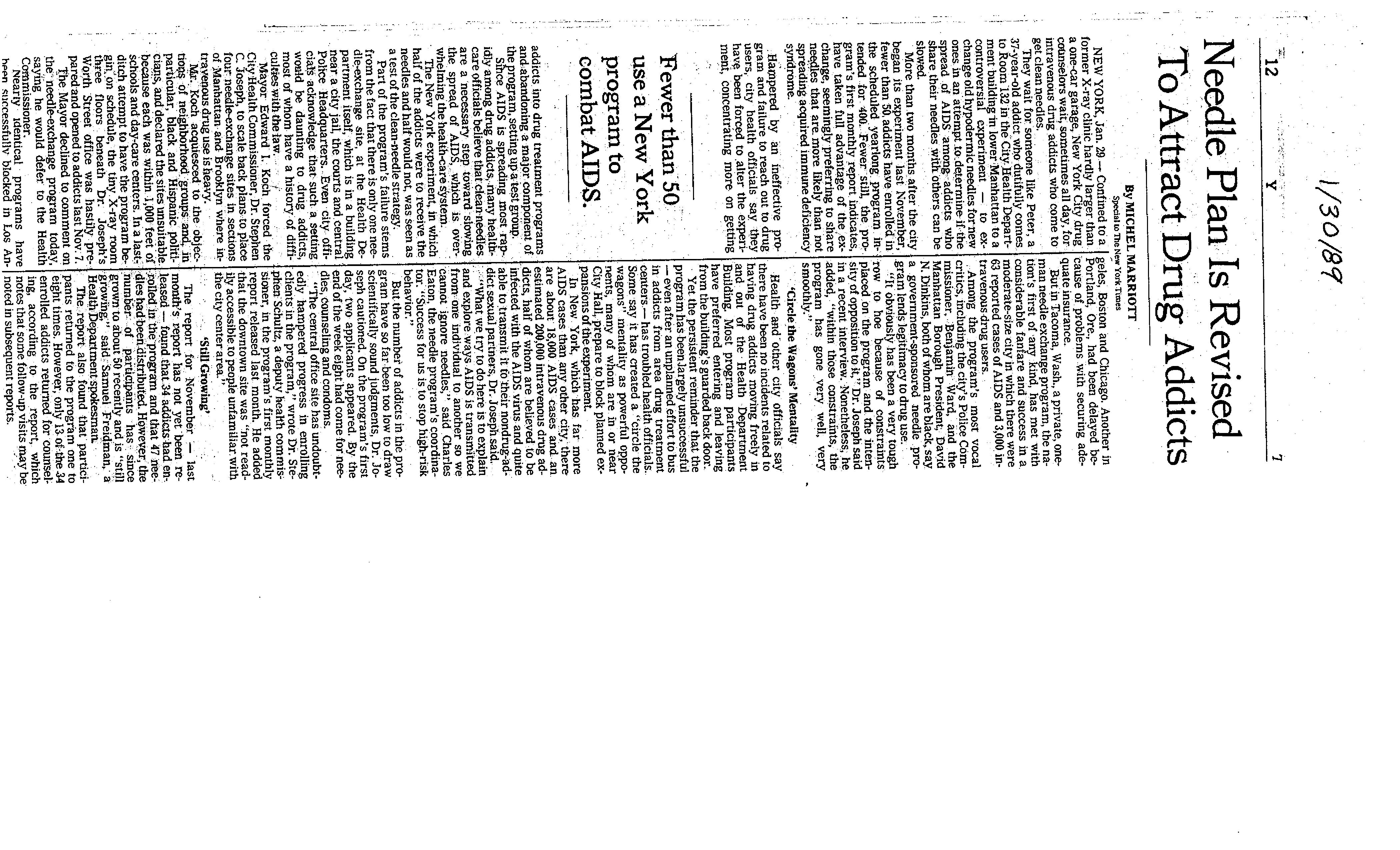

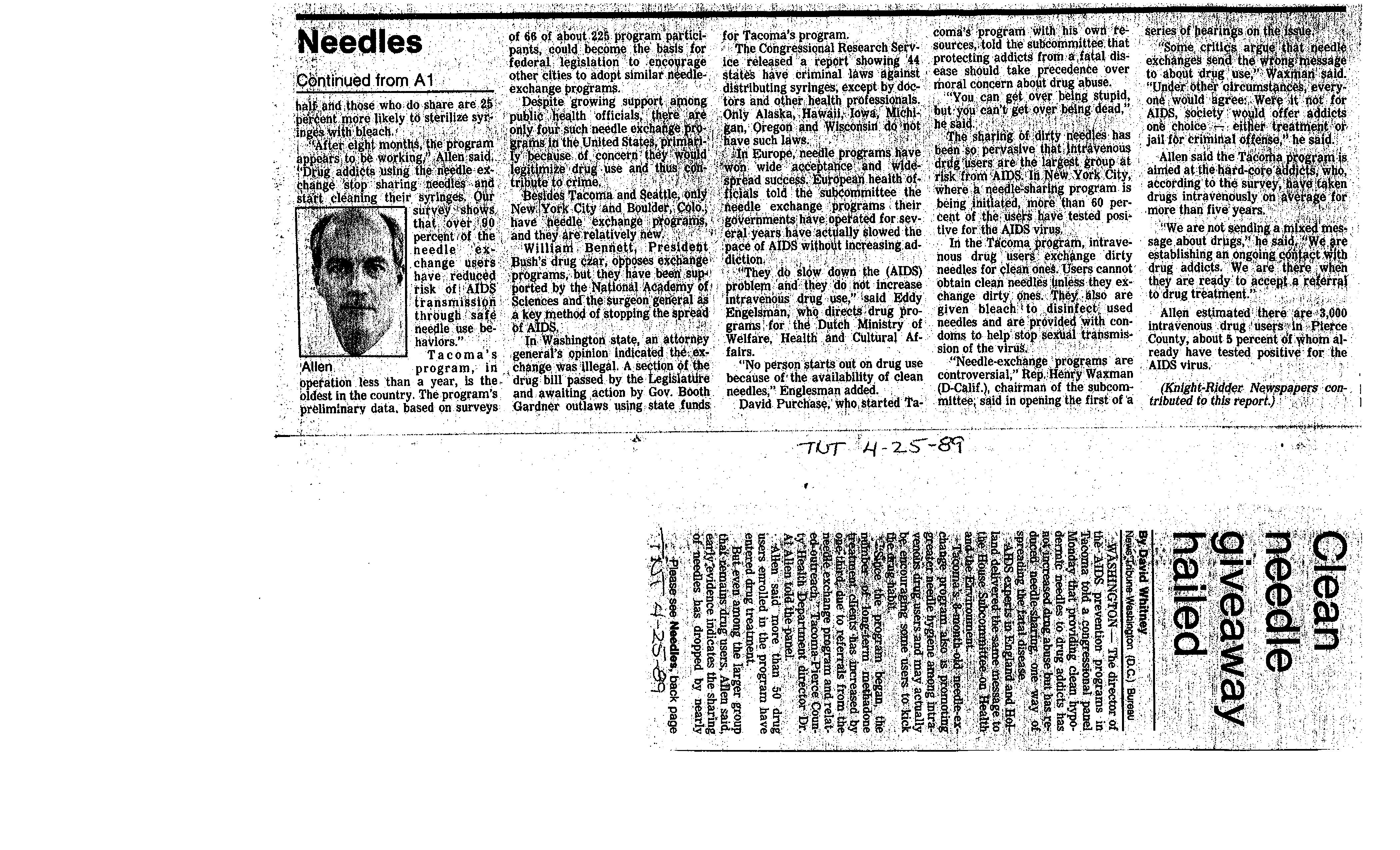

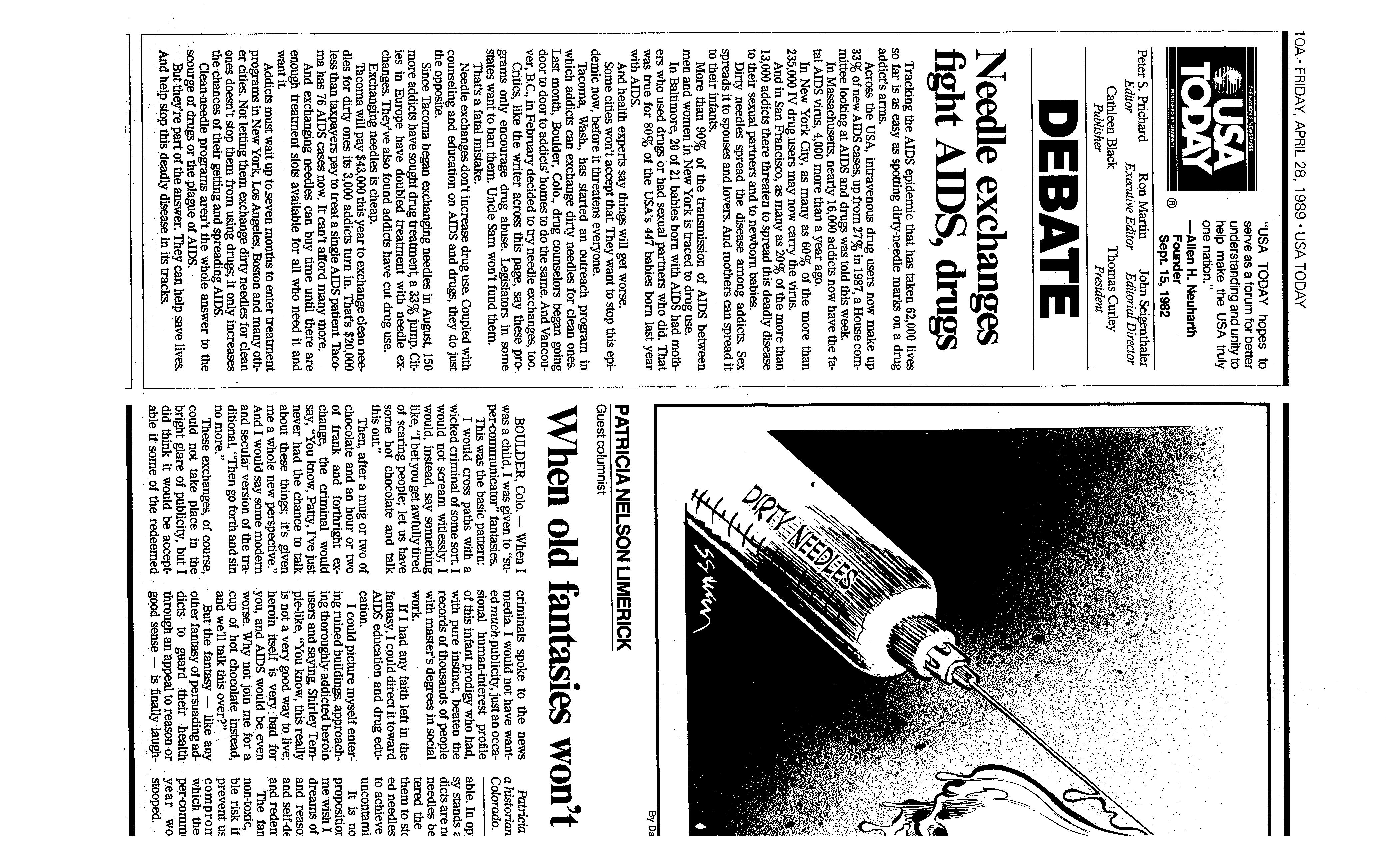

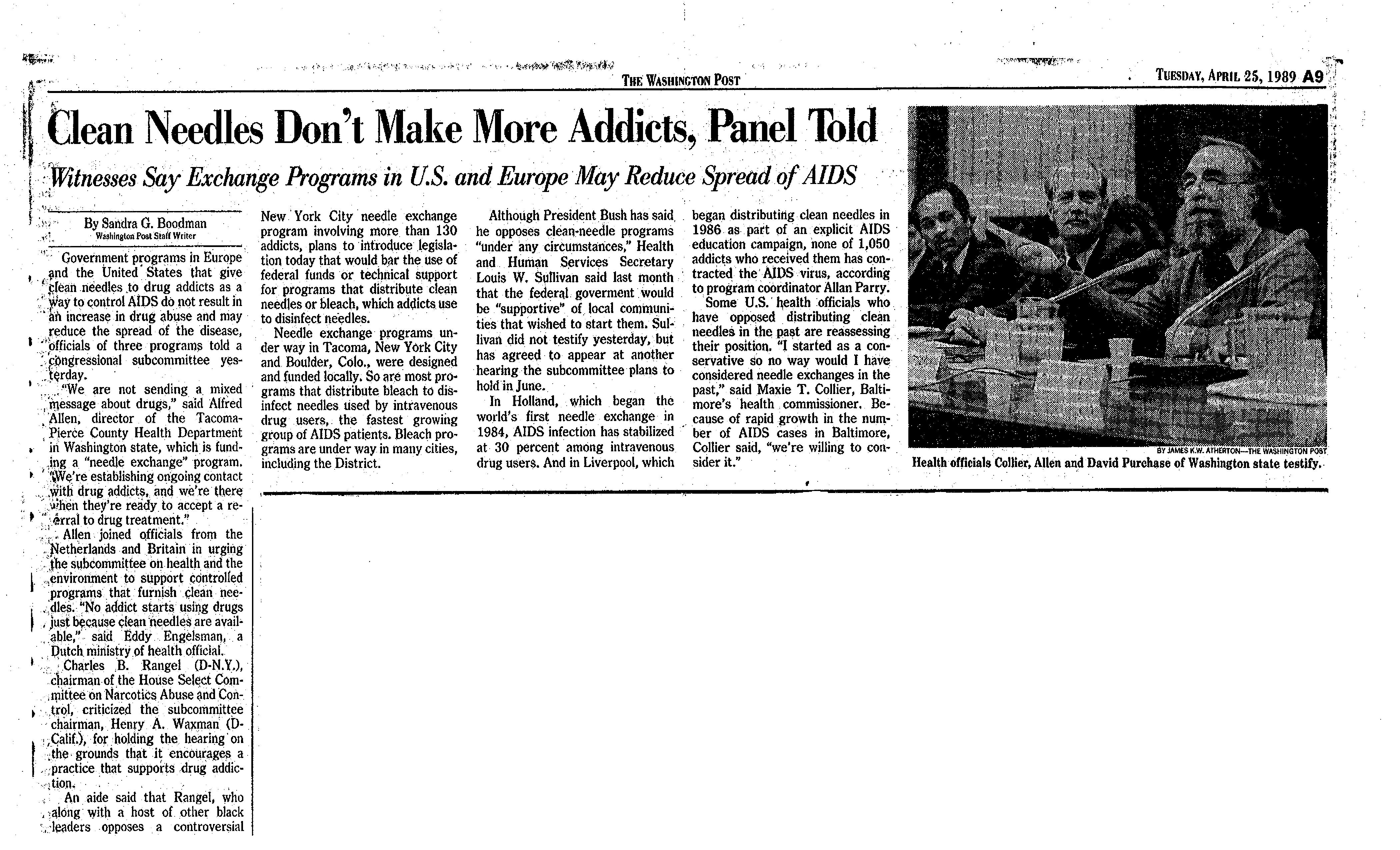

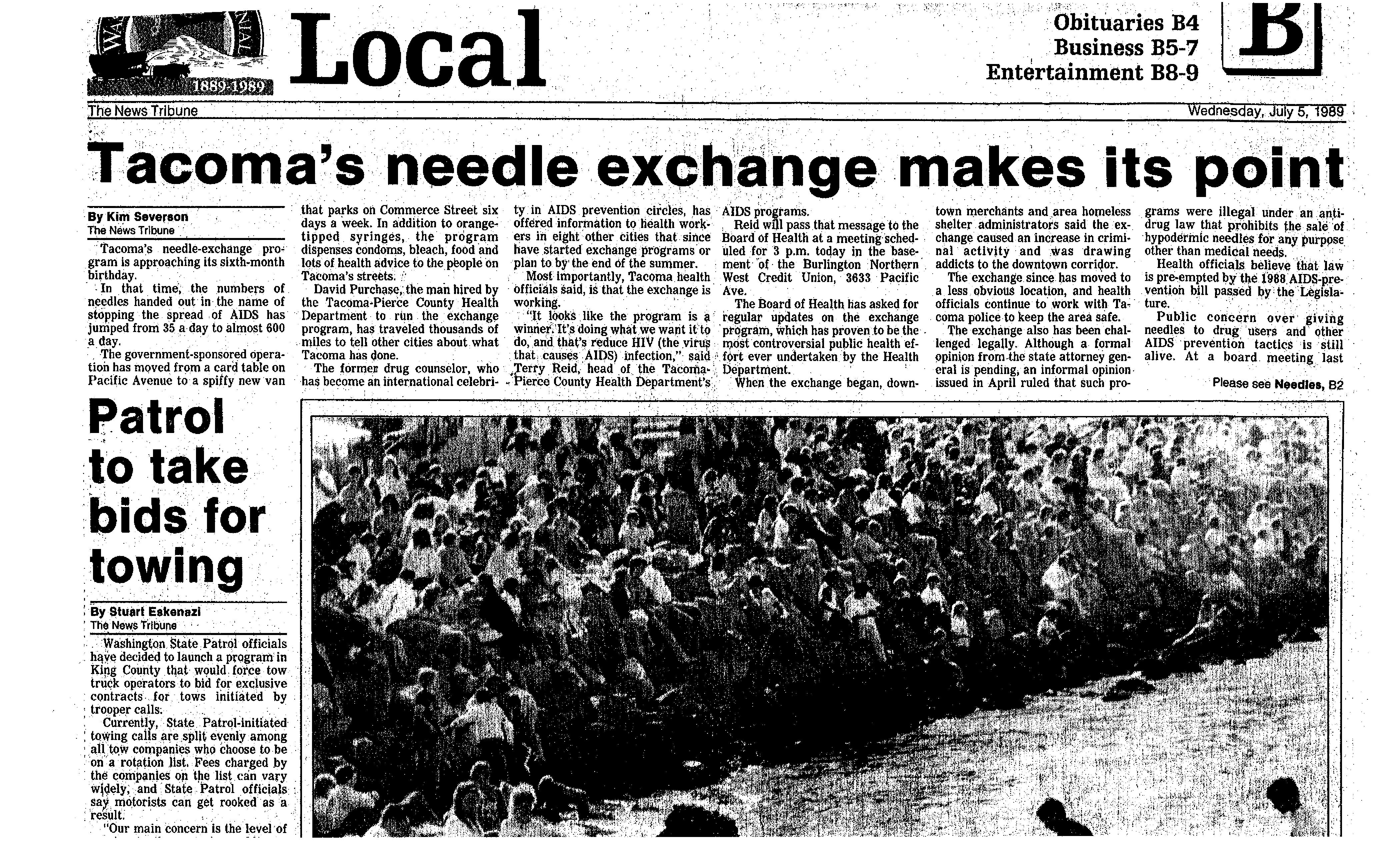

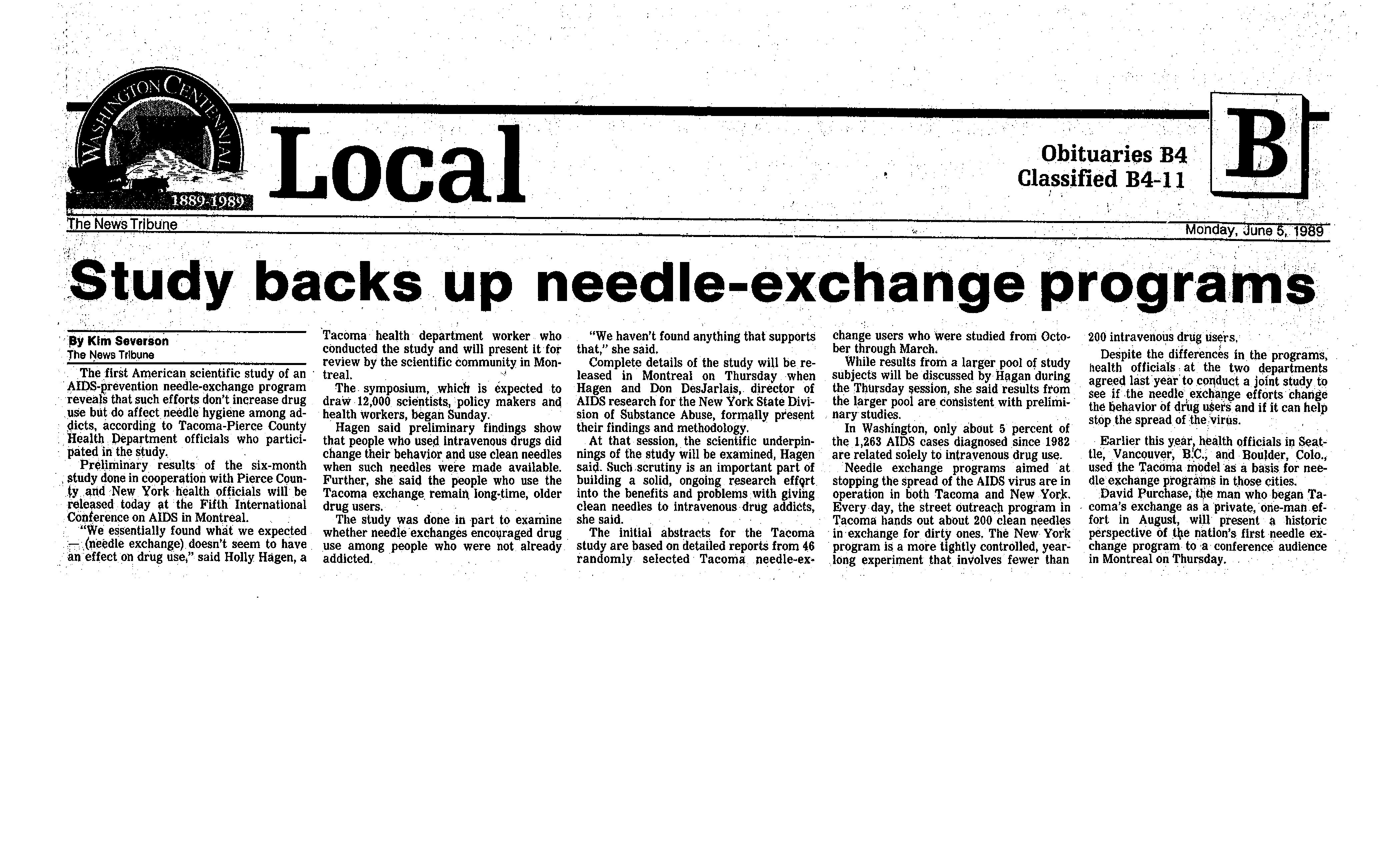

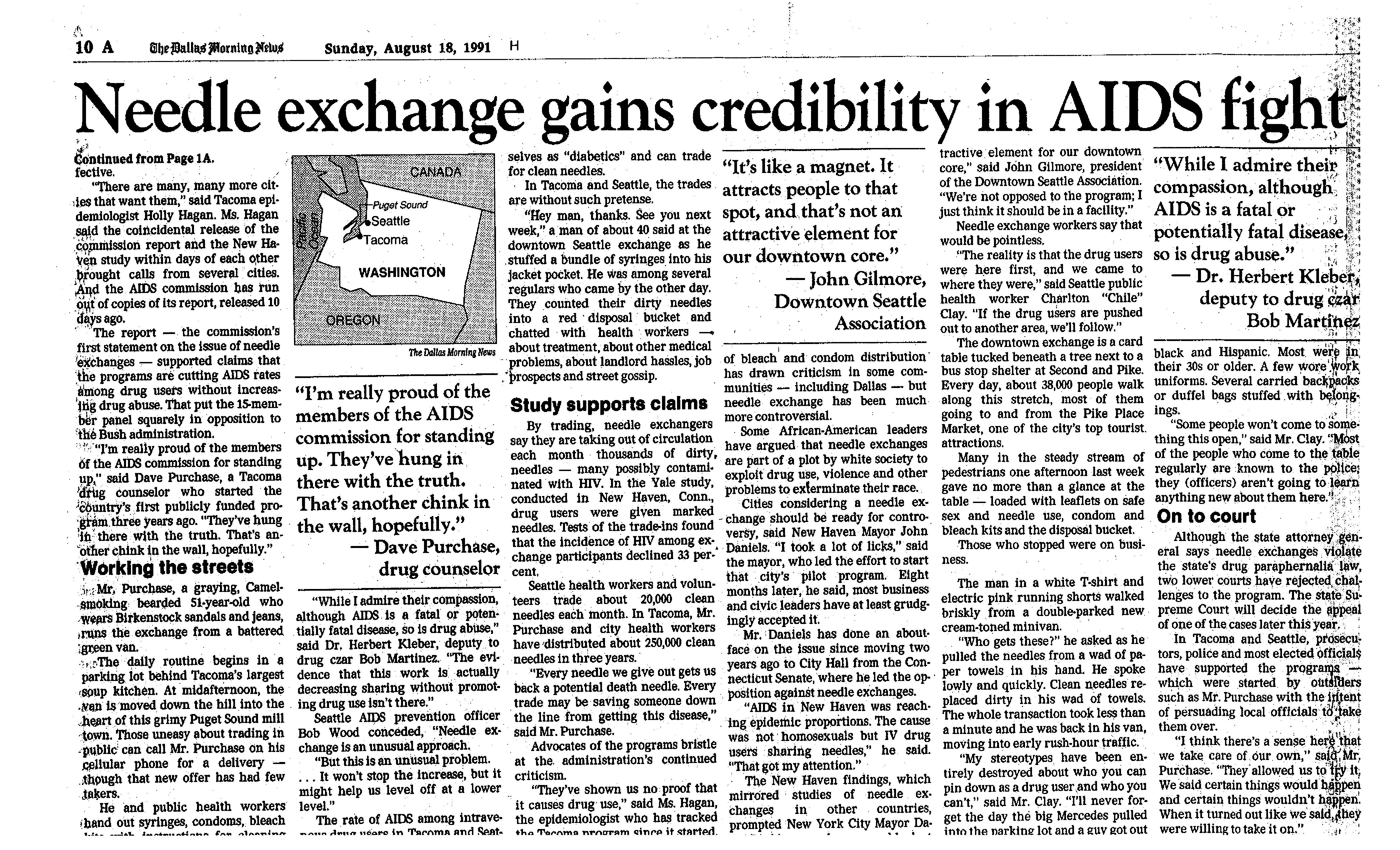

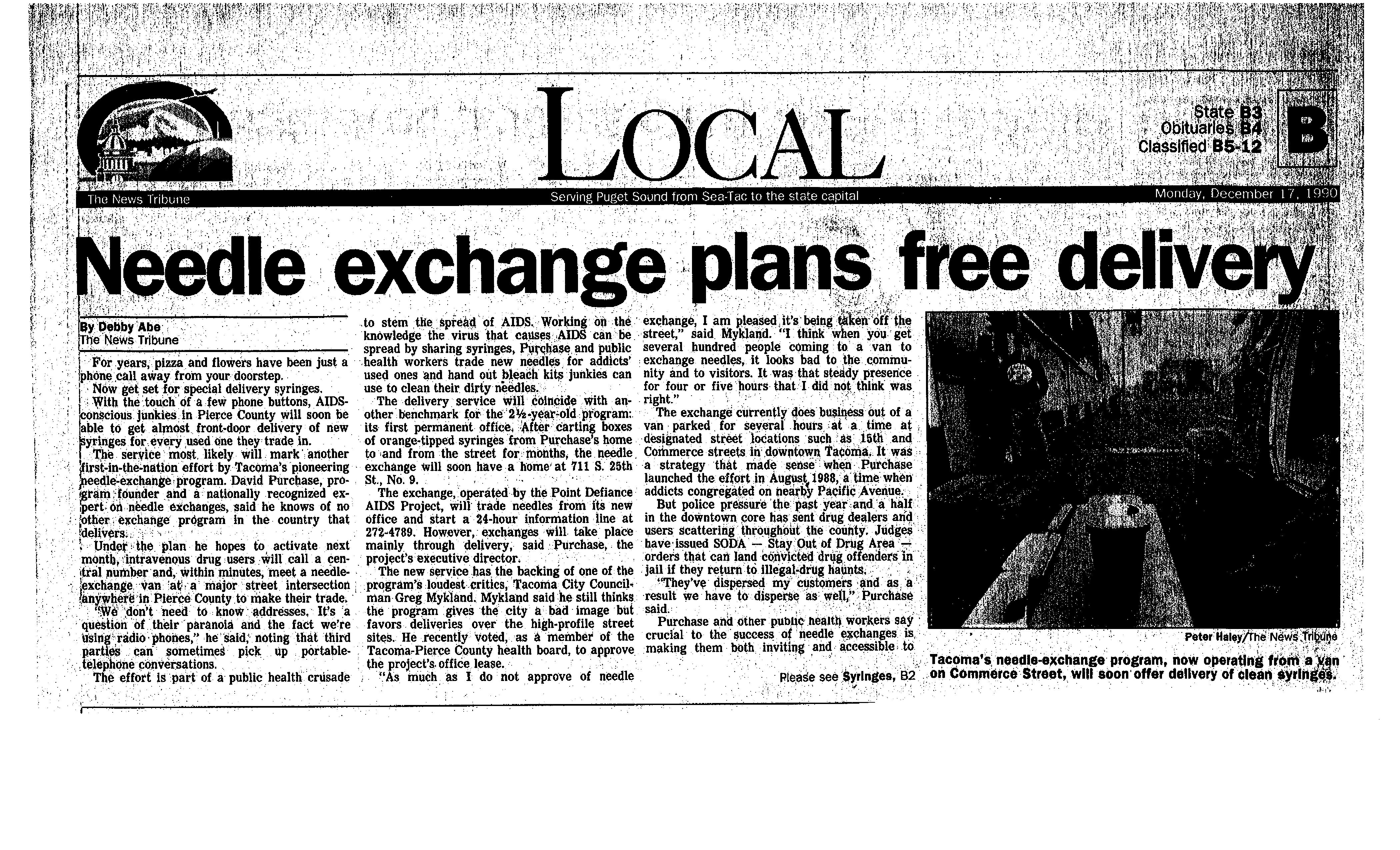

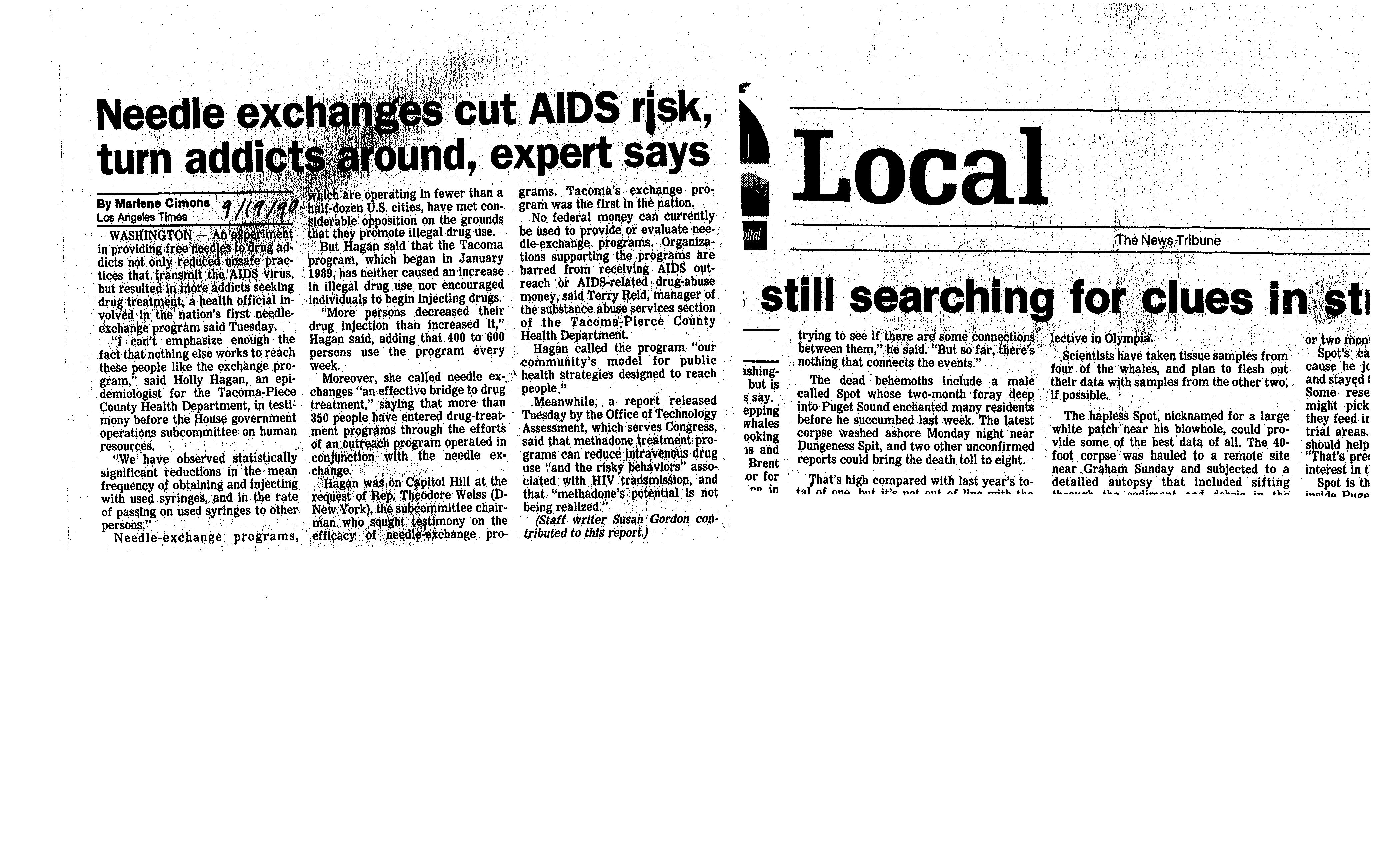

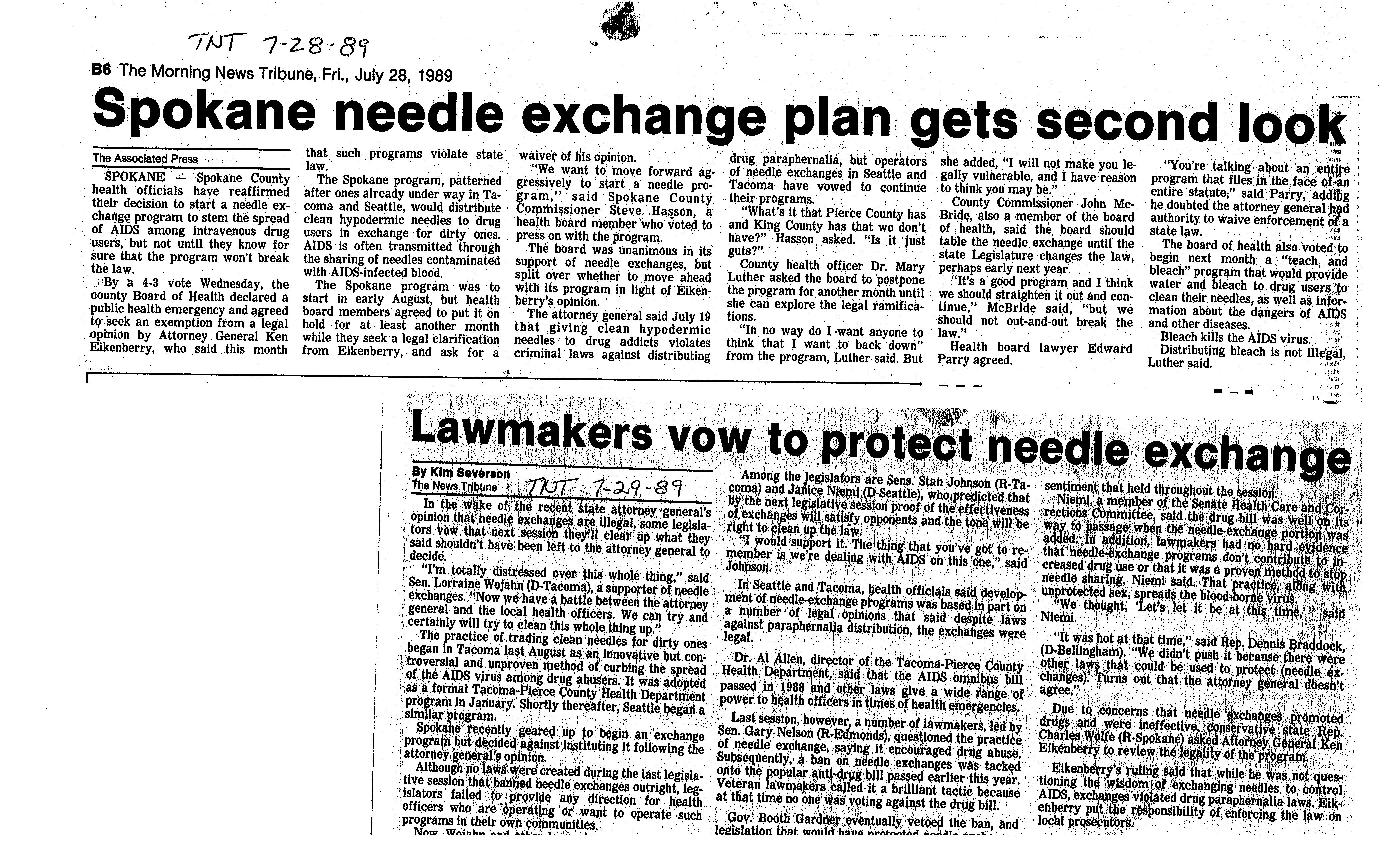


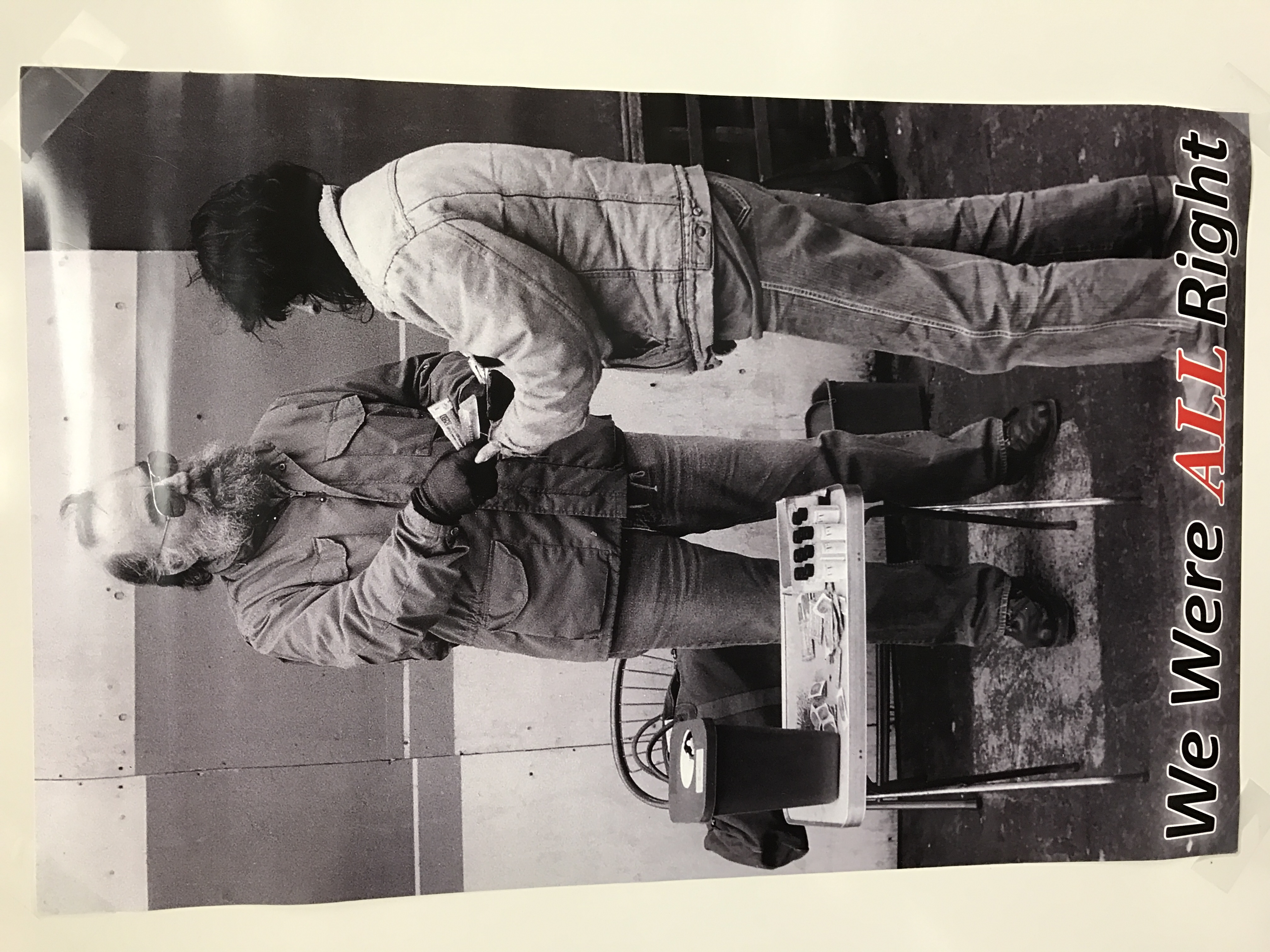

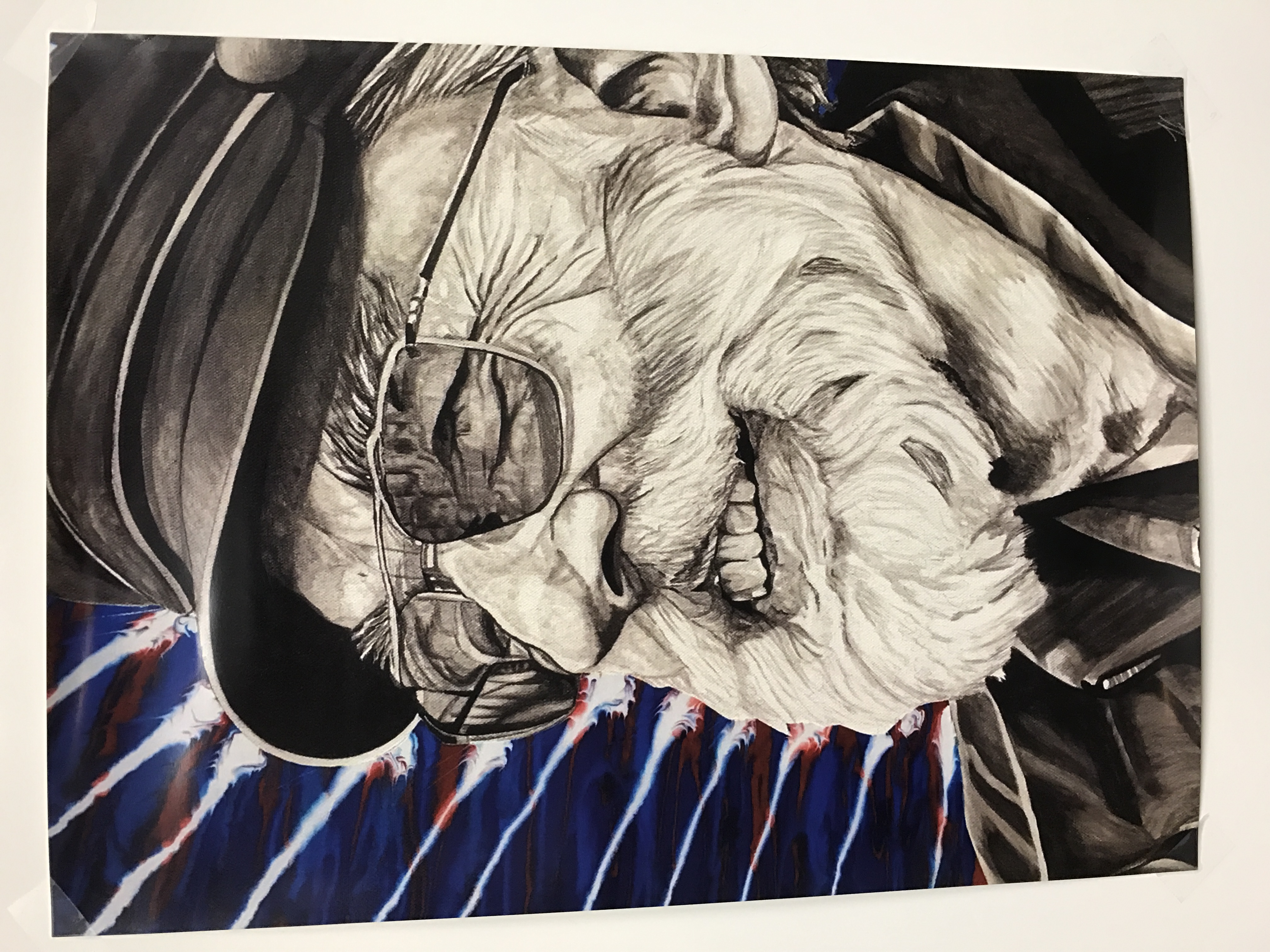

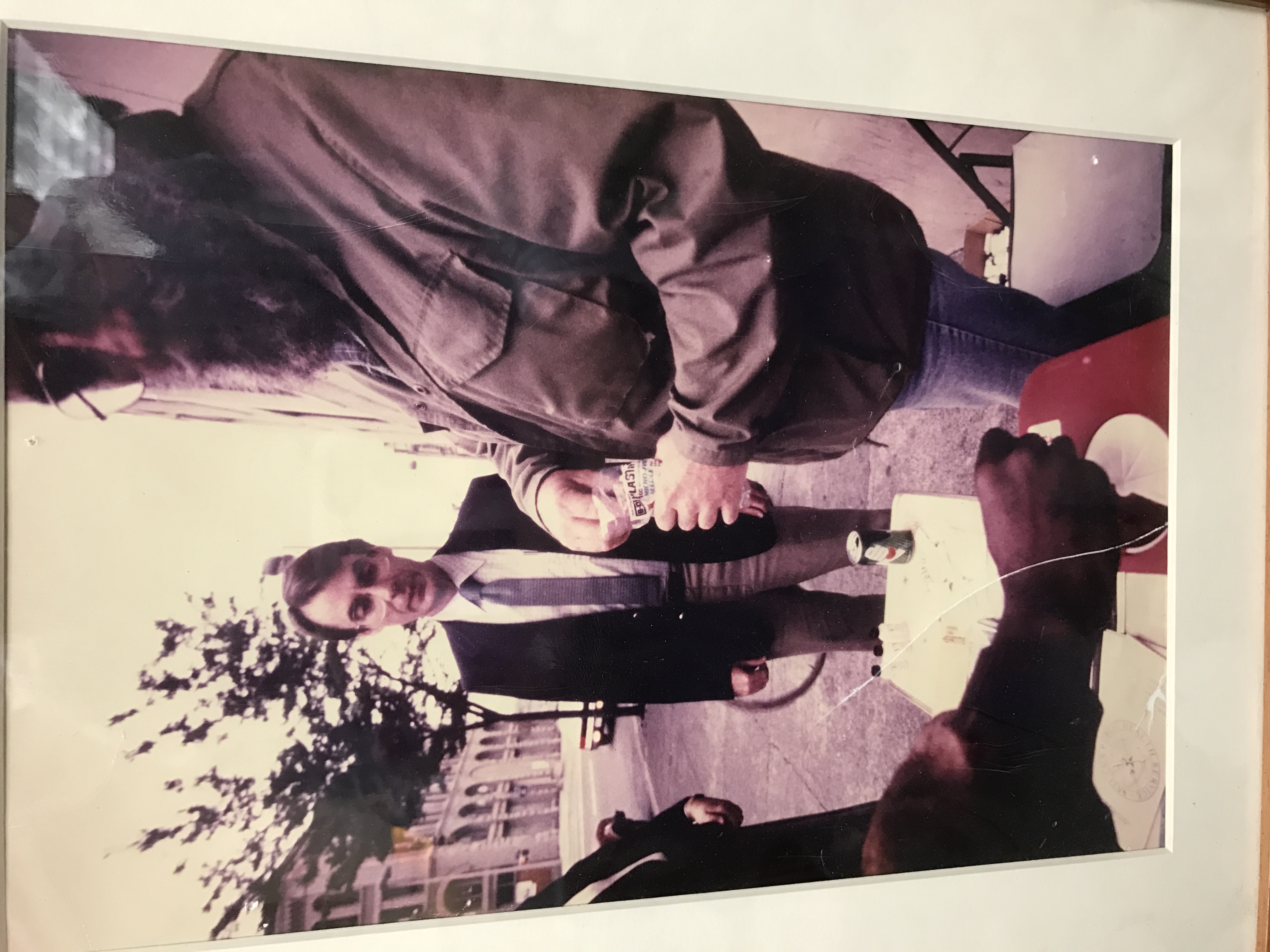

Supplement: Additional file 1: — Dave Purchase Press Stories Collection Appendix. (DOC 78422 kb) [file 12954_2017_178_MOESM1_ESM.doc]
